# Silver(I) Complexes Bearing S-Alkyl Thiosalicylic Acid Derivatives: DNA/BSA Binding and Antitumor Activity In Vitro and In Vivo

**DOI:** 10.3390/pharmaceutics17101340

**Published:** 2025-10-16

**Authors:** Jovana Marinković, Milena Jurišević, Marina Jovanović, Miloš Milosavljević, Ivan Jovanović, Snežana Jovanović Stević, Marina Vesović, Miloš Nikolić, Nikola Nedeljković, Ana Živanović, Dušan Tomović, Andriana Bukonjić, Gordana Radić, Nevena Gajović

**Affiliations:** 1 Faculty of Medicine of the Military Medical Academy, University of Defence, Crnotravska 17, 11000 Beograd, Serbia; jovana.eko@gmail.com; 2 Center for Molecular Medicine and Stem Cell Research, Faculty of Medical Sciences, University of Kragujevac, Svetozara Markovića 69, 34000 Kragujevac, Serbia; marina_jovanovic@rocketmail.com (M.J.); ivanjovanovic77@gmail.com (I.J.); gajovicnevena@yahoo.com (N.G.); 3 Department of Pharmacy, Faculty of Medical Sciences, University of Kragujevac, Svetozara Markovića 69, 34000 Kragujevac, Serbia; snezanaj@kg.ac.rs (S.J.S.); milos.nikolic@fmn.kg.ac.rs (M.N.); nikola.nedeljkovic@fmn.kg.ac.rs (N.N.); ana.zivanovic@fmn.kg.ac.rs (A.Ž.); dusantomovic88@hotmail.com (D.T.); andriana.bukonjic@hotmail.com (A.B.); vasic_gordana@yahoo.com (G.R.); 4 Department of Otorhinolaryngology, Faculty of Medical Sciences, University of Kragujevac, 34000 Kragujevac, Serbia; 5 Department of Pathology, University Medical Center Kragujevac, 34000 Kragujevac, Serbia; m.milosavljevic77@gmail.com; 6 Faculty of Medicine, University of East Sarajevo, Studentska 5, 73300 Foca, Bosnia and Herzegovina

**Keywords:** silver(I) complex, BSA, DNA, cytotoxicity, apoptosis, breast cancer

## Abstract

**Background/Objectives:** In recent years, silver complexes have shown strong antibacterial, antifungal, and antitumor activity with high selectivity toward cancer cells. Their cytotoxic effects are mainly linked to apoptosis induction, DNA damage, and enzyme inhibition, while the antitumor activity of silver(I) complexes with S-alkyl thiosalicylic acid derivatives remains unexplored. **Methods:** Silver(I) complexes with S-alkyl derivatives of thiosalicylic acid (**C1**–**C5**) were obtained through the direct reaction of silver(I) nitrate, the corresponding ligand of thiosalicylic acid, and a sodium hydroxide solution. The interactions between the complexes and CT-DNA/BSA were studied using UV-Vis, fluorescence spectroscopy, and molecular docking studies. The cytotoxic capacity of the newly synthesized complexes was assessed by an MTT assay. **Results**: Complexes **C1**–**C5** exhibited strong cytotoxicity against murine and human breast (4T1, MDA-MB-468), colon (CT26, HCT116), and lung (LLC1, A549) cancer cell lines. The **C3** complex significantly diminished tumor progression in an orthotropic mammary carcinoma model while demonstrating good systemic tolerance. **Conclusions**: The tested complex **C3** triggered apoptosis in 4T1 cells by altering the delicate balance between pro- and anti-apoptotic Bcl-2 family members, increasing reactive oxygen species (ROS) levels, and reducing mitochondrial membrane depolarization. Moreover, the **C3** arrested the 4T1 cell cycle in G0/G1 phase, decreasing the expression of cyclin D3 and increasing the expression of p16, p21, and p27.

## 1. Introduction

Numerous limitations in the use of cisplatin have led recent studies on antitumor drugs to increasingly focus on the development of metal complexes that include alternative metal ions and novel organic ligands [1]. Silver ions have attracted particular interest due to their well-documented antimicrobial, antiseptic, and anti-inflammatory properties, combined with relatively low toxicity to humans [2]. However, a major limitation of silver salts lies in their high solubility under physiological conditions, which leads to dissociation and compromises their therapeutic efficacy [3]. Therefore, significant efforts are being directed toward designing silver-based metal complexes that exhibit enhanced stability in human serum. Among the promising ligands for coordination are salicylic acid and its derivatives, which have long been recognized for their antibacterial, antifungal, and antitumor properties [4].

Thiosalicylic acid is an organosulfur compound containing both carboxyl and sulphydryl functional groups. The combination of “hard” and “soft” donor atoms enables various coordination modes with various metal ions [5]. Thiosalicylic acid exhibits a range of pharmacological effects, including anti-inflammatory, antioxidant, and analgesic properties, and numerous derivatives of this compound are utilized in medicine. Farnesyl thiosalicylic acid has attracted attention due to its unique mechanism of action. By mimicking the structure of the RAS protein (rat sarcoma protein), this drug binds to its site on the cell membrane, thereby preventing the activation of the RAS signaling pathway [6]. Apart from the fact that the molecular mechanism of farnesylthiosalicylic acid forms the basis for newer principles of oncological therapy, these derivatives represent a promising strategy for the development of new cytotoxic agents targeting various types of cancer [7]. Previously published studies revealed that the investigated salicylic acid derivative affects the synthesis of maspin by stimulating systemic nitric oxide (NO) production, which slows the disease progression in breast cancer patients [8,9]. However, there is strong evidence to suggest that NSAIDs, especially salicylates, exhibit chemopreventive effects and reduce the risk of colorectal cancer [10,11]. Aspirin stimulates the degradation of Iκβα, an inhibitor of nuclear factor kappa beta (NF-κβ) transcription, which mediates the increased synthesis of antioxidants and induces apoptosis of colorectal cancer cells [12]. The tested zinc complex with the S-propyl derivative of thiosalicylic acid showed exceptional activity, inhibiting breast cancer growth and promoting apoptosis by a Bcl-2-controlled mitochondrial pathway. This effect was also supported by G1/S cell cycle arrest of tumor growth following upregulation of cyclin-dependent kinase inhibitors p16, p21, and p27 and downregulation of STAT-3, c-Myc, and cyclin D3 [13]. The same ligand, the S-propyl derivative of thiosalicylic acid, complexed with a platinum ion achieves a promising effect in murine B-cell leukemia lymphoma cells by modulating the activity of two signaling pathways, STAT3 and NF-B, and by sequentially altering the ratio of pro- and anti-apoptotic proteins, ultimately leading to the induction of caspase-3-dependent apoptosis [14].

In recent years, silver complexes have been extensively investigated as potential antibacterial, antitumor, and antifungal agents in numerous studies [15]. The results of antifungal tests indicate the superiority of silver complexes over standard treatments against voriconazole-resistant fungi, which may be associated with the inhibition of efflux pump activity in resistant strains [16]. The antitumor activity of silver-based terpyridine complexes has been evaluated in multiple cell lines and shown to induce cell death through activation of the mitochondrial apoptotic pathway [17]. The efficacy of silver complexes with 1,10-phenanthroline and thiosemicarbazone ligands has been demonstrated in human lung carcinoma cells (A449) and human breast carcinoma cells (MDA-MB-231 and MCF-7). These complexes exhibited pronounced cytotoxic capacity with a minor reduction in the viability of human non-tumor breast cells (MCF-10A). The observed cytotoxic effects are primarily attributed to the induction of apoptosis and cell cycle arrest in the sub-G1 phase, as well as increased production of free radicals and reduced mitochondrial membrane potential [18]. Furthermore, silver complexes with bidentate NHC (*N*-heterocyclic carbene) ligands significantly reduced the viability of human breast carcinoma (MCF-7) and human colon adenocarcinoma (DLD1) cells [19]. Notably, the Ag(NHC)_2_AgBr_2_ exerts its antitumor effect through multiple mechanisms: it inhibits Trx-R (thioredoxin reductase), which leads to increased production of free oxygen radicals and the promotion of apoptosis; it inhibits topoisomerase I, leading to DNA molecule damage; and it inhibits PARP-1 (Poly [ADP-ribose] polymerase 1) while quickly and selectively inhibiting glycolysis in tumor cells. These findings suggest that the mechanism of action of the tested complex is distinct from that of cisplatin and that it is more selective towards tumor cells than non-tumor cells. The cytotoxic potency of the investigated silver complexes on over 16 cell lines was found to be comparable to or greater than that of cisplatin, with an improved selectivity profile [20]. Silver complexes with triphenylphosphine and semicarbazone ligands have demonstrated a significant reduction in the viability of human breast cancer cells (MDA-MB-231 and MCF-7) and prostate cancer cells (DU-14). Their cytotoxic effects were up to 15 times greater than those of cisplatin, with a notably higher selectivity index towards tumor cells compared to non-tumor human prostate cells [21]. A silver thiosulfate complex has been shown to decrease the viability of human breast cancer cells (MCF-7), arresting the cell cycle in the G1 phase and reducing glutathione levels with increased production of oxygen-free radicals. This complex also exhibited a high degree of selectivity towards tumor cells [22]. The cytotoxic potential of silver complexes with phenanthroline derivatives as ligands was confirmed on human prostate cancer and ovarian cancer tumor cells resistant to cisplatin [23]. In recent years, silver complexes with ligands derived from non-steroidal anti-inflammatory drugs have garnered considerable attention. Silver complexes with naproxen, niflumic acid, and picoline have reduced the viability of human colorectal cancer cells (HT-29), lung cancer (A-549), and breast cancer (MDA-MB-453). The tested complexes induced apoptosis of colon cancer tumor cells, which is a consequence of mitochondrial membrane depolarization and activation of the caspase cascade [24]. Similarly, silver complexes with diclofenac and niflumic acid trigger the apoptotic death of human breast and colon cancer tumor cells, arrest the cell cycle in the sub-G1 phase, and activate caspases [25]. Two binuclear silver complexes with tolfenamic acid and 2-methylpyridine ligands have exhibited enhanced cytotoxicity against human breast cancer cells (MDA-MB-453), surpassing the efficacy of carboplatin and showing superior selectivity towards tumor cells. The strong apoptotic potential of these complexes is associated with the arrest of the cell cycle in the G1 phase, increased expression of pro-apoptotic markers (Bax, caspase-3, caspase-9, and p53), and downregulation of the anti-apoptotic protein bcl-2. In addition, these complexes increase the release of free oxygen radicals and NO, contributing to mitochondrial membrane damage [26]. To the author’s knowledge, the antitumor activities of the silver(I) complex with S-alkyl derivatives of thiosalicylic acid have not been tested yet.

Taking all into consideration, the present study evaluates the potential ability of newly synthetized silver(I) complexes with S-alkyl derivatives of thiosalicylic acid to suppress the viability of breast, colon, and lung cancer cells in vitro. In addition, the antitumor effects of the complex with the S-propyl derivative of thiosalicylic acid against murine breast cancer cells (4T1) in vitro and in vivo were established. This complex diminished breast cancer progression through suppressing cell proliferation and inducing caspase-dependent apoptosis. The binding interactions of new compounds and CT-DNA/BSA were investigated using different spectroscopic methods and molecular docking studies.

## 2. Materials and Methods

### 2.1. Chemistry

#### Reagents and Instruments

All chemical reagents were of analytical grade, obtained from a commercial supplier (Sigma-Aldrich, St. Louis, MO, USA), and used without further purification. The structural characterization of the synthesized silver(I) complexes was performed by elemental microanalysis and by using IR, NMR, and molar conductivity measurements.

Elemental microanalysis was performed by standard methods on a Vario III CHNS Elemental Analyzer (Elemental Analysen-systeme GmbH, Langenselbold, Germany). For the infrared spectra, a PerkinElmer FTIR 31725-X spectrophotometer (Waltham, MA, USA) was employed using the KBr pellet technique. The nuclear magnetic resonance spectra of the synthesized compounds were recorded using a Varian Gemini-200 NMR spectrophotometer (Palo Alto, CA, USA). The molar conductance values of freshly prepared solutions of silver(I) complexes were measured by Eutech Instruments CON 700 conductometer (Singapore).

Ethidium bromide (EB) [3,8-diamino-5-ethyl-6-phenylphenanthridinium bromide], Hoechst 33258 (Hoe), calf thymus DNA (CT-DNA), and bovine serum albumin (BSA) were obtained from Sigma-Aldrich, Burlington, MA, USA, and used without further purification. A stock solution of calf thymus DNA (CT-DNA) was prepared in 0.01 M phosphate-buffered saline (PBS, pH 7.4; Sigma-Aldrich, Burlington, MA, USA). The UV absorbance ratio at 260 nm and 280 nm (A_620_/A_280_) was approximately 1.8–1.9, confirming the absence of protein contamination. The concentration of CT-DNA was determined spectrophotometrically using a molar extinction coefficient of 6600 M^−1^ cm^−1^ at 260 nm [27,28]. Stock solution of bovine serum albumin (BSA) was prepared in 0.01 M PBS (pH 7.4), and the concentration was maintained at 2 μM. The concentration ranges employed in the UV-Vis and fluorescence experiments were carefully selected to ensure clear linearity and adequate spectral changes, with all utilized concentration ranges proving suitable for the precise and reliable determination of binding and quenching parameters.

The interactions between complexes and CT-DNA/BSA were studied using the UV-Vis spectroscopy on a PerkinElmer Lambda 35 (PerkinElmer, Shelton, CT, USA) double-beam spectrophotometer equipped with thermostated 1.00 cm quartz Suprasil cells, as well as the fluorescence spectroscopy on an RF-1501 PC spectrofluorometer (Shimadzu, Kyoto, Japan).

Mouse breast cancer (4T1), human breast cancer (MDA-MB-468), mouse colon cancer (CT26), human colon cancer (HCT-116), mouse lung cancer (LLC1), human lung cancer (A549), mouse mesenchymal stem cells (mMSC), and human fibroblast cell lines (MRC-5) were used in this study and were consistently cultured in complete Dulbecco’s Modified Eagle Medium (DMEM) (Sigma-Aldrich, St. Louis, MO, USA) in incubator (5% CO_2_) under standard conditions. Complete DMEM was Dulbecco’s Modified Eagle Medium supplemented with 10% fetal bovine serum (FBS), 1 mmol/L penicillin–streptomycin, 1 mmol/L mixed nonessential amino acids, and 2 mmol/L L-glutamine (Sigma-Aldrich, St. Louis, MO, USA). All cancer cell lines and MRC-5 cell line were obtained from American Type Culture Collection (ATCC, Manassas, VA, USA), but mMSC was manufactured by Gibco (Middlesex County, MA, USA).

### 2.2. Syntheses

#### 2.2.1. General Procedure for the Synthesis of Bidentate Ligands—S-Alkyl Derivatives of Thiosalicylic Acid (**L1**–**L5**)

The bidentate ligands, S-alkyl derivatives of thiosalicylic acid (alkyl = methyl (**L1**), ethyl (**L2**), propyl (**L3**), butyl (**L4**), benzyl (**L5**)), were prepared according to the previously described procedure by alkylation of thiosalicylic acid using the corresponding alkyl halides in alkaline water–ethanol solution [29]. For this study, thiosalicylic acid derivatives with varying side chain lengths were selected for complex synthesis to evaluate their influence on biological activity. The length of the alkyl chain particularly affects lipophilicity, which in turn influences the compound’s ability to cross cell membranes and to establish relevant interactions with key macromolecules. In addition, increasing the alkyl chain length may enhance biological activity up to a certain threshold, beyond which activity may decline sharply.

#### 2.2.2. General Procedure for the Synthesis of Silver(I) Complexes with S-Alkyl Derivatives of Thiosalicylic Acid (**C1**–**C5**)

Silver(I) complexes with S-alkyl derivatives of thiosalicylic acid were obtained by direct reaction of silver(I) nitrate, the corresponding ligand of thiosalicylic acid, and sodium hydroxide solution.

##### Preparation of the Complex [Ag_2_(S-Methyl-Thiosal)_2_] (**C1**)

To the solution obtained by dissolving 0.0841 g (0.5 mmol) of S-methyl derivative of thiosalicylic acid in 5 mL of distilled water, 5 mL of 0.1 M sodium hydroxide solution is slowly added. The solution of ligand prepared in this manner is then gradually added to a solution obtained by dissolving 0.0849 g (0.5 mmol) of silver(I) nitrate in 5 mL of distilled water. The reaction mixture was left protected from light at room temperature for another 2 h with stirring on a magnetic stirrer. The resulting white precipitate of complex **C1** is separated by filtration, washed with distilled water, and dried in air. Yield: 0.0816 g (59.34%). Anal. Calc. for C_16_H_14_O_4_S_2_Ag_2_ (Mr = 550.15): C, 34.93; H, 2.57; S, 11.66; found: C, 34.78; H, 2.52; S, 11.60; molar conductivity: 12.86 S∙cm^2^∙mol^−1^. IR (KBr, cm^−1^): 3429, 2915, 1588, 1568, 1537, 1526, 1425, 1369, 1282, 1162, 1063, 967, 837, 753, 704, 693, 653. ^1^H NMR (200 MHz, DMSO-*d*_6_, *δ* ppm): 2.38 (s, 6H, CH_3_), 7.11–7.85 (m, 8H, Ar). ^13^C NMR (50 MHz, DMSO-*d*_6_, *δ* ppm): 14.9 (CH_3_), 124.9–139.7 (Ar), 171.5 (COO^−^).

##### Preparation of the Complex [Ag_2_(S-Ethyl-Thiosal)_2_] (**C2**)

The complex [Ag_2_(S-ethyl-thiosal)_2_] (**C2**) was prepared as described in Section Preparation of the complex [Ag_2_(S-Methyl-Thiosal)_2_] (**C1**) using the S-ethyl derivative of thiosalicylic (0.0911 g, 0.5 mmol) instead of the S-methyl derivative of thiosalicylic acid. Yield: 0.0891 g (61.64%). Anal. Calc. for C_18_H_18_O_4_S_2_Ag_2_ (Mr = 578.20): C, 37.39; H, 3.14; S, 11.09; found: C, 37.18; H, 3.15; S, 11.29; molar conductivity: 11.36 S∙cm^2^∙mol^−1^. IR (KBr, cm^−1^): 3432, 2924, 1588, 1570, 1537, 1435, 1370, 1278, 1163, 1061, 977, 838, 737, 689, 655. ^1^H NMR (200 MHz, DMSO-*d*_6_, *δ* ppm): 1.29 (t, 6H, CH_3_), 2.90 (q, 4H, CH_2_), 7.09–7.75 (m, 8H, Ar). ^13^C NMR (50 MHz, DMSO-*d*_6_, *δ* ppm): 13.7 (CH_3_), 26.0 (CH_2_), 123.9–136.9 (Ar), 172.5 (COO^−^).

##### Preparation of the Complex [Ag_2_(S-Propyl-Thiosal)_2_] (**C3**)

The complex [Ag_2_(S-propyl-thiosal)_2_] (**C3**) was prepared as described in Section Preparation of the complex [Ag_2_(S-Methyl-Thiosal)_2_] (**C1**) using the S-propyl derivative of thiosalicylic (0.0981 g, 0.5 mmol) instead of S-methyl derivative of thiosalicylic acid. Yield: 0.0914 g (60.31%). Anal. Calc. for C_20_H_22_O_4_S_2_Ag_2_ (Mr = 606.25): C, 39.62; H, 3.66; S, 10.58; found: C, 39.57; H, 3.64; S, 10.54; molar conductivity: 7.75 S∙cm^2^∙mol^−1^. IR (KBr, cm^−1^): 3443, 2954, 1590, 1576, 1547, 1454, 1380, 1366, 1278, 1162, 1060, 898, 835, 740, 689, 655. ^1^H NMR (200 MHz, DMSO-*d*_6_, *δ* ppm): 0.98 (t, 6H, CH_3_), 1.52 (m, 4H, CH_2_), 2.92 (t, 4H, CH_2_), 7.18–7.95 (m, 8H, Ar). ^13^C NMR (50 MHz, DMSO-*d*_6_, *δ* ppm): 13.2 (CH_3_), 21.9 (CH_2_), 27.4 (CH_2_), 125.1–137.1 (Ar), 172.9 (COO^−^).

##### Preparation of the Complex [Ag_2_(S-Butyl-Thiosal)_2_] (**C4**)

The complex [Ag_2_(S-butyl-thiosal)_2_] (**C4**) was prepared as described in Section Preparation of the complex [Ag_2_(S-Methyl-Thiosal)_2_] (**C1**) using the S-butyl derivative of thiosalicylic (0.1051 g, 0.5 mmol) instead of S-methyl derivative of thiosalicylic acid. Yield: 0.1011 g (63.76%). Anal. Calc. for C_22_H_26_O_4_S_2_Ag_2_ (Mr = 634.31): C, 41.66; H, 4.13; S, 10.11; found: C, 41.52; H, 4.06; S, 10.03; molar conductivity: 8.88 S∙cm^2^∙mol^−1^. IR (KBr, cm^−1^): 3425, 2958, 1589, 1575, 1548, 1434, 1380, 1368, 1278, 1164, 1060, 896, 837, 744, 688, 655. ^1^H NMR (200 MHz, DMSO-*d*_6_, *δ* ppm): 0.93 (t, 6H, CH_3_), 1.40 (m, 4H, CH_2_), 1.65 (m, 4H, CH_2_), 2.82 (t, 4H, CH_2_), 7.23–8.05 (m, 8H, Ar). ^13^C NMR (50 MHz, DMSO-*d*_6_, *δ* ppm): 13.1 (CH_3_), 20.7 (CH_2_), 28.9 (CH_2_), 34.7 (CH_2_), 124.1–136.9 (Ar), 171.9 (COO^−^).

##### Preparation of the Complex [Ag_2_(S-Benzyl-Thiosal)_2_], (**C5**)

The complex [Ag_2_(S-benzyl-thiosal)_2_] (**C5**) was prepared as described in Section Preparation of the complex [Ag_2_(S-Methyl-Thiosal)_2_] (**C1**) using the S-benzyl derivative of thiosalicylic (0.1221 g, 0.5 mmol) instead of S-methyl derivative of thiosalicylic acid. Yield: 0.1027 g (58.47%). Anal. Calc. for C_28_H_22_O_4_S_2_Ag_2_ (Mr = 702.34): C, 47.88; H, 3.16; S, 9.13; found: C, 47.72; H, 3.13; S, 9.02; molar conductivity: 13.85 S∙cm^2^∙mol^−1^. IR (KBr, cm^−1^): 3399, 3059, 1589, 1573, 1550, 1433, 1377, 1279, 1162, 1062, 840, 779, 744, 695, 654. ^1^H NMR (200 MHz, DMSO-*d*_6_, *δ* ppm): 4.23 (s, 4H, CH_2_), 7.15–8.03 (m, 18H, Ar and bz). ^13^C NMR (50 MHz, DMSO-*d*_6_, *δ* ppm): 34.9 (CH_2_), 126.1–137.5 (bz), 124.9–138.0 (Ar), 172.4 (COO^−^).

### 2.3. Absorption Spectroscopic Studies

UV-Vis absorption spectroscopy was employed to investigate the relative binding affinities of complexes **C1**–**C5** toward CT-DNA. All DNA-binding studies were conducted in 0.01 M phosphate-buffered saline (PBS, pH 7.40) at 25 °C. Complex–DNA solutions were prepared by maintaining a constant complex concentration (8 μM) while gradually increasing the concentration of CT-DNA stock solution (2.13 mM). To eliminate absorbance from CT-DNA, identical amounts of DNA were added to both the test and reference solutions prior to spectral recording [30].

Intrinsic binding constants (*K*_b_) were calculated using the equation to determine the DNA-binding affinities of the complexes (1) [31]:

[DNA]/(*ε*_a_ − *ε*_f_) = [DNA]/(*ε*_b_ − *ε*_f_) + 1 [*K*_b_(*ε*_b_ − *ε*_f_)] (1)

[DNA] represents the concentration of DNA; apparent extinction coefficient, *ε*_a_, was calculated as the ratio of the observed absorbance to the concentration of the complex (A_obs_/[complex]); and *ε*_b_ and ε_f_ correspond to the extinction coefficients of the complex in the fully bound and free forms, respectively. *K*_b_ was determined from the ratio of the slope to the intercept obtained from the corresponding linear plots.

### 2.4. Fluorescence Quenching Measurements

To investigate the binding interactions of the complexes with CT-DNA, fluorescence measurements were carried out using EB–CT-DNA and Hoe–CT-DNA solutions (8.52 × 10^−5^ M), to which varying concentrations of the complexes **C1**–**C5** (for EB) and **C4** (for Hoe) were gradually added in the range of (0–6.82) × 10^−5^ M. Emission spectra for EB were recorded between 550 and 750 nm after excitation at 527 nm, while for Hoe, they were recorded in the range of 300 to 600 nm with an excitation at 346 nm. All samples were prepared in phosphate-buffered saline (PBS, pH 7.4). Stern–Volmer Equation (2) [32] was used to interpret the fluorescence quenching data. *I*_0_ and *I* represent the fluorescence intensities without and with the quencher (**C1**–**C5**), respectively; [Q] is the quencher concentration; and Stern–Volmer quenching constant, *K*_sv_, was obtained from the slope of the plot of *I*_0_/*I* against [Q].

*I*_0_/*I* =1 + *K*_sv_[Q] (2)

### 2.5. Viscosity Measurements

Viscosity measurements were performed to evaluate the interaction of **C2** and **C4** with DNA. The flow time was measured with a digital stopwatch. For each concentration, six replicate measurements were taken, and the average flow time was used for analysis. The data were presented as (*η*/*η*_0_)^1/3^ against *r*, where *η* is the viscosity of DNA in the presence of complex, and *η*_0_ is the viscosity of DNA alone in the buffered solution. The viscosity value was calculated from the observed flow time of DNA-containing solution (*t*) corrected for the flow time of buffer alone (*t*_0_), *η* = (*t* − *t*_0_)/*t*_0_.

### 2.6. Albumin-Binding Studies

The protein binding study was carried out using tryptophan fluorescence quenching experiments with BSA. A constant BSA concentration of 2 µM was maintained, while the concentrations of complexes **C1**–**C5** were varied from 0.4 to 8 µM, resulting in a molar ratio of r = [BSA]/[complex] ranging from 0.2 to 4. Fluorescence emission spectra were recorded between 300 and 500 nm, with excitation at 295 nm.

The quenching constant *K*_sv_ was determined based on the Stern–Volmer relationship, as described by Equation (3) [32]:

*I*_0_/*I* = 1 + *k*_q_τ_0_[Q] = 1 + *K*_sv_[Q] (3)

In this equation, *I*_0_ and *I* refer to the fluorescence intensities of BSA measured without and with the quencher (complex), τ_0_ represents the average fluorescence lifetime of free BSA (∼10^−8^ s), and [Q] indicates the quencher concentration.

Parameters such as association binding constant (*K*) and the number of binding sites per albumin (*n*) were obtained from Scatchard Equation (4) [33]:

(Δ*I*/*I_0_*)/[Q] = *nK* − *K*Δ*I*/*I_0_* (4)

To minimize inner filter, both intensities of fluorescence originating from excitation and emission light absorption and re-absorption were corrected by employing Equation (5) [34]; *I_cor_* and *I_obs_* are the corrected and observed fluorescence intensities, respectively, and *Aex* and *Aem* represent the absorbance of the metal complex at the excitation and emission wavelengths, respectively.

*I_cor_* = *I_obs_* · e^(*Aex* + *Aem*)/2^ (5)

### 2.7. In Silico Molecular Docking Studies

Molecular docking was enforced for silver(I) complexes to assess and compare their theoretical and experimental binding affinity for DNA and BSA using the AutoDock 4.2 software [35]. The geometries of all five tested complexes were initially pre-optimized using the semiempirical PM3 (Parameterized Model 3) method and subsequently refined through energy minimization employing the Hartree–Fock method with the 3–21G basis set. Both stages of the optimization were performed with the Gaussian computational package implemented in Chem3D Ultra 7. The crystal structures of DNA with an intercalative binding anticancer agent (PDB ID: 1Z3F), canonical B-DNA dodecamer (PDB ID: 1BNA), and BSA (PDB ID: 4F5S) were retrieved from the Protein Data Bank (http://www.rcsb.org/pdb, accessed on 1 September 2025.). Structures of target were prepared by removing water molecules, co-crystallized ligands, and nonessential heteroatoms. For the docking simulation with DNA molecules, the search area was defined as a grid box with dimensions of 50 × 50 × 50 Å^3^ separated by 0.375 Å for short sequences of DNA, 60 × 74 × 120 Å^3^, separated by 0.375 Å, for B-form sequence d(CGCGAATTCGCG), and 126 × 126 × 126 Å^3^ for BSA. In docking simulation, flexible silver(I) complexes were fit into the rigid structure of target molecules. Molecular docking was performed using the Lamarckian Genetic Algorithm with default parameters, including a population size of 150, a maximum number of energy evaluations and generations of 2.5 million and 27.000, with a mutation rate of 0.02 and a crossover rate of 0.8. Ten docking runs have been generated as binding conformations, and the best-docked conformations of tested silver(I) complexes were visualized using PyMol 2.4.1 [36].

### 2.8. MTT Assay

In order to evaluate cytotoxic capacity of silver(I) complexes with S-alkyl derivatives of thiosalicylic acid (**C1**–**C5**), MTT assay was performed as previously described in detail [37]. In 96-well microplates, we seeded cancer and non-cancer cells, more exactly 5 × 10^3^ cells per well in 100 μL of complete DMEM (Sigma-Aldrich, St. Louis, MO, USA). After 24 h, cells were grown in the presence of examined complexes **C1**–**C5**, cisplatin (CDDP), or complete DMEM alone for next 24 or 48 h (Sigma-Aldrich, St. Louis, MO, USA). Tested concentration range for all metal-based substances was 3.9–500 μM. After incubation with MTT solution (5 mg/mL; pH 7.2) for 4 h, optical density was determined using Zenyth 3100 microplate multimode detector (Salzburg, Austria). All experiments were performed three times, each in triplicate, and the IC_50_ values as well as selectivity indexes (IC_50_ for mMSC or MRC-5/IC_50_ for mouse or human cancer cells) were calculated.

### 2.9. Evaluation of Breast Cancer Development In Vivo

Ethics Committee of the Faculty of Medical Sciences of the University of Kragujevac, Serbia, approved all experiments (01-5301/11). ARRIVE guidelines and EU Directive 2010/63/EU for animal experiments were followed. Experimental animals, BALB/C female mice (8–10 weeks old), were housed under standard conditions (temperature-controlled environment, a 12 h light–dark cycle, fed ad libitum, and observed daily) throughout whole duration of experiment. Animals were inoculated with 4T1 cells (3 × 10^4^) into the fourth mammary fat pad as previously described [38]. Animals were randomly assigned to experimental or control groups, and after palpable tumors were detected, the pharmacological treatments were started. Female BALB/C mice intraperitoneally received either **C3** (3 mg/kg), **C3** (6 mg/kg), CDDP (6 mg/kg), or vehicle in distilled water with 0.05% DMSO (Sigma-Aldrich, St. Louis, MO, USA) (untreated group) in five single doses every third day (8 animals per group). Using electronic calipers (Moore and Wright, Sheffield, UK), the sizes of primary 4T1 mammary tumors were morphometrically evaluated [39]. On the 30th day of experiment, animals were sacrificed, blood was collected (for determination of serum levels of ALT, AST, urea, and creatinine), and tissue sections (liver and kidney) were stained with H&E for further analysis. Liver or kidney sections were analyzed by two observers, and the injury levels were determined as previously reported [38].

### 2.10. Colony Formation Assay

Cancer 4T1 cells were seeded in a six-well plate (500 cells per well) and incubated with **C3** (0.5 µM) or DMEM only for 14 days. After fixation, the colonies were stained using crystal violet.

### 2.11. Analysis of Cell Death

4T1 cells were treated with 0.5 µM of **C3** or grown in DMEM only (control 4T1 cells) for 24 h and stained with Annexin-V-FITC and propidium iodide (PI) (BD Pharmingen, San Diego, CA, USA) as previously described [26]. Quantification of cellular death was measured using FACSCalibur Flow Cytometer (BD Biosciences, San Jose, CA, USA) and FlowJo software (version 10.7.2; Tree Star Inc. Ashland, OR, USA).

### 2.12. Cell Cycle Distribution

After reaching optimal confluency, 4T1 cells were allowed to grow in the presence of 0.5 µM of **C3** or DMEM only (control 4T1 cells) for next 24 h. According to manufacturer’s recommendation, cells were stained with Vybrant^®^ DyeCycle^TM^ Ruby stain (Thermo Fisher Scientific, Waltham, MA, USA) in order to perform cell cycle analysis. The analysis was carried out on FACSCalibur Flow Cytometer (BD Biosciences, San Jose, CA, USA), and data were analyzed using FlowJo software (version 10.7.2; Tree Star Inc. Ashland, OR, USA).

### 2.13. Flow Cytometry Analyses

Flow cytometry was performed on 4T1 cells treated with 0.5 µM of **C3** for 24 h or untreated cells. After treatment, cells were incubated with antibodies specific for Bax (Abcam Cambridge, Cambridge, UK), Bcl-2 (Abcam Cambridge, UK), BclXL (Thermo Fisher Scientific, Waltham, MA, USA), MCL-1 (Thermo Fisher Scientific, Waltham, MA, USA) caspase 3 (Abcam Cambridge, UK), Ki-67 (eBioscience, San Diego, CA, USA), cyclin D3 (Abcam Cambridge, UK), p21 (Abcam Cambridge, UK), p16 (Abcam Cambridge, UK), and p27 (Abcam Cambridge, UK) and secondary antibodies as previously described [37,40]. FACSCalibur Flow Cytometer (BD Biosciences, San Jose, CA, USA) was used for flow cytometry, and the data were analyzed using FlowJo (version 10.7.2; Tree Star Inc. Ashland, OR, USA).

### 2.14. The Measurement of Mitochondrial Membrane Potential and ROS Levels

After 24 h of **C3** treatment, 4T1 cells were stained with appropriate dyes, followed by flow cytometry analysis using FACS Calibur Flow Cytometer (BD Biosciences, San Jose, CA, USA). Mitochondrial membrane potential stained by Rhodamine-123 (Sigma-Aldrich, St. Louis, MO, USA), TMRE (Abcam, Cambridge, UK), and cellular ROS levels were stained by DC-FHDA (Sigma-Aldrich, St. Louis, MO, USA).

### 2.15. Scratch Assay

In order to determine migratory capacity of **C3**-treated 4T1 cells, scratch assay was performed as previously described [38]. Once 4T1 cells formed a confluent monolayer, an injury was made, and first representative images were taken immediately. Further 4T1 cells were exposed to **C3** (0.5 µM) or DMEM only (untreated cells), and observation under inverted optical microscope (Olympus, Tokyo, Japan) was made after 4 h, 12 h, and 24 h. Scratch width was quantified by ImageJ (version 1.54.0 NIH, Madison, WI, USA).

### 2.16. Statistical Analysis

All these data were presented as average ± S.D. Student’s *t*-test or Mann–Whitney rank-sum test was used based on the normality of data distribution. The difference between more than two groups was determined by one-way ANOVA or Kruskal–Wallis test. A significance level of <0.05 was considered statistically significant.

## 3. Results and Discussion

### 3.1. Synthesis and Chemical Characterization

**Scheme 1:**
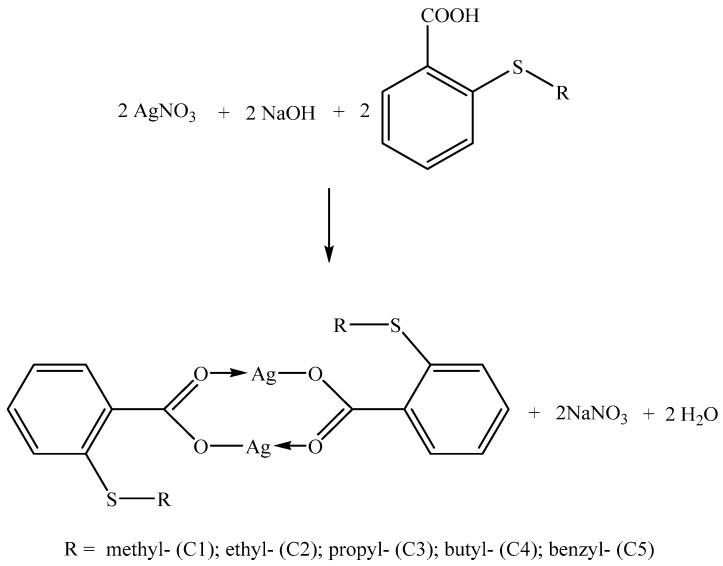
Synthesis of silver(I) complexes with S-alkyl derivatives of thiosalicylic acid (**C1**–**C5**).

Silver(I) complexes (**C1**–**C5**) were obtained by direct reaction of silver(I) nitrate and the corresponding S-alkyl derivative of thiosalicylic acid in a molar ratio of 1:1, with the addition of an aqueous solution of sodium hydroxide (Scheme 1).

The elemental microanalysis results obtained for carbon, sulfur, and hydrogen are in agreement with theoretically calculated values, which confirmed the composition of the newly synthesized complexes.

Molar conductivity was measured using solutions of the synthesized complexes at a concentration of 10^−3^ mol/L in dimethyl sulfoxide. The obtained values of specific conductivity were converted into molar conductivity using the concentration of the solutions. Very low values of molar conductivity (<50 S∙cm^2^∙mol^−1^) indicate the non-electrolyte nature of the complex solutions and confirm the absence of nitrate anions both inside and outside the coordination sphere [41].

To determine the coordination mode of metal ions to the ligand, the infrared spectra of the complexes were recorded and compared with those of the ligands. The IR bands of the carboxyl group are very significant and useful for determining the coordination type of ligands and metal ions within the synthesized coordination compounds. The disappearance of the O-H stretching band (2500 to 3000 cm^−1^) in the carboxyl group of the newly synthesized complexes provides clear evidence of coordination between the carboxylate anion and the silver ion. Namely, in the infrared spectrum of the complex, two intense bands are observed, the asymmetric stretching ν_asym_ (COO^−^) in the region around ~1540 cm^−1^ and the symmetric stretching ν_sym_ (COO^−^) in the region around ~1370 cm^−1^, indicating the presence of a carboxylate anion. The shift of the asymmetric valence vibration C=O to lower frequency values, compared to free ligands, suggests bond weakening due to delocalization of π-electrons and coordination with the metal ion. The presence ν(C-S) band in the complexes (653 cm^−1^ for **C1**, 655 cm^−1^ for **C2**, 655 cm^−1^ for **C3**, 655 cm^−1^ for **C4**, and 654 cm^−1^ for **C5**) at almost the same wavelength as observed in the IR spectra ligands (652 cm^−1^ for **L1**, 651 cm^−1^ for **L2**, 653 cm^−1^ for **L3**, 651 cm^−1^ for **L4**, 652 cm^−1^ for **L5**) indicates the definite absence of coordination of the silver ion to the sulfur atom.

The mode of coordination of the carboxylate anion to the metal ion can also be assumed based on the empirical correlation between the positions ν_asym_ COO^−^ and ν_sym_ COO^−^ of carboxylate groups, as well as the frequency difference between them (∆*ν*). Generally, ∆*ν* values greater than about 350 cm^−1^ indicate monodentate binding, values between 200 < ∆*ν* < 350 cm^−1^ suggest an anisobidentate binding (the state between monodentate and bidentate), and ∆*ν* values less than 200 cm^−1^ point to binuclear bidentate bridging complexes [42]. The infrared data for our complexes (Table 1) show that S-alkyl derivatives act as bidentate bridging ligands in the formation of binuclear Ag(I) complexes, coordinating to the metal ion via the oxygen atom of the carboxyl group and thus suggesting greater stability of the complex.

Aromatic protons are observed in a broad range of chemical shifts from 7.15 to 8.17 ppm for the ligand and from 7.09 to 8.05 ppm for the complex, respectively, suggesting that coordination did not alter the position or shape of the signal. By adding the ligand to a sample of the previously recorded ^1^H NMR spectrum of the complex, we found no significant chemical shift of the alkyl group protons, ruling out coordination via the sulfur atom. The absence of a hydrogen atom of the carboxyl group in the ^1^H NMR spectra of the silver complexes indicates that the carboxyl group is not protonated after coordination.

The carbon atoms of the aromatic ring are expected to be found at the highest chemical shifts, ranging from 123.9 to 138.0 ppm. The signals of the carbon atoms of the alkyl groups are also located at their characteristic chemical shifts, which match the literature data and support the proposed structure of the synthesized complex. Notably, the chemical shifts remained unchanged during a 24 h period, suggesting that the complex remains intact and exhibits enhanced stability, potentially mitigating the risks of silver ion dissociation and uncontrolled reactivity in vivo.

Moreover, similar coordination modes were observed in previously published studies involving ligands with analogous chemical structures [43]. In the solid state, silver(I) carboxylates predominantly form bridged dimers, as confirmed by the crystal structures of silver(I) benzoate and p-hydroxybenzoate [42]. In the study of Azócar and colleagues, the structure of the complex of silver with salicylic acid was confirmed by X-ray diffraction, which showed that the coordination occurs through the oxygen atoms of the carboxyl group and that the complex exists as a dimeric structure Ag_2_(Sal)_2_ [44].

### 3.2. DNA-Binding Studies

#### 3.2.1. Absorption Spectroscopic Studies

DNA is a crucial therapeutic target for many anticancer metal-based agents, so the obtained information during the study of drug-DNA interaction is of important interest for understanding the mechanism of drug action and for the design of an effective antitumor drug. Electronic absorption spectroscopy is a commonly used method to study the binding mode of metal complexes to DNA [45].

**Figure 1:**
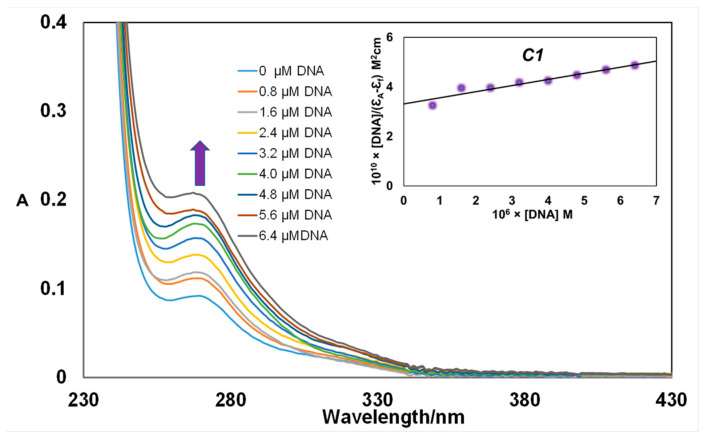
Absorption spectra of **C1** at 25 °C in PBS buffer upon addition of CT-DNA. [complex] = 8 × 10^−6^ M, [DNA] = (0–6.4) × 10^−6^ M. The arrow shows the change in the absorbance with the increase in DNA concentration. Inset: plot of [DNA]/(εA–εf) vs. [DNA].

Figure 1 shows the UV-Vis spectra of **C1** with and without CT-DNA. The addition of CT-DNA induces a hyperchromic effect and the emergence of a new band around 270 nm, suggesting non-covalent interactions with DNA [34]. Similar behavior was observed for the other examined complexes **C2**–**C5** (Figure S1, Supplementary Materials).

The binding constants, *K*_b_, were determined to quantitatively compare the binding affinity of the complexes to DNA. The ratio of slope and intercept was obtained in a plot of [DNK]/(*ε*_a_ − *ε*_f_) vs. [DNK] (insert plot in Figure 1 and Figure S1, Supplementary Materials), and calculated *K*_b_ values are given in Table 2. The high binding constants (10^4^–10^5^ M^−1^) indicate strong binding affinity of the complexes for CT-DNA, following the reactivity order: **C5** > **C4** > **C3** > **C2** > **C1**. Variations in DNA-binding strength are attributed to differences in ligand side groups. Compared to ethidium bromide (*K*_b_ = (1.23 ± 0.07) × 10^5^ M^−1^) [46], complexes **C3**–**C5** exhibit higher binding affinities, while **C1** and **C2** show lower binding affinities.

Calculated negative values of free energies (Δ*G*) for **C1**–**C5** (Table 2) using Equation (6) [47,48] indicate that the binding of complexes **C1**–**C5** to CT-DNA occurs spontaneously.

Δ*G* = −*RT* ln*K*_b_ (6)

#### 3.2.2. Viscosity Measurements

The viscosity of DNA solutions is measured to determine whether metal complexes interact with DNA through intercalation. Intercalation typically leads to an extension of the DNA strand, as the base pairs are pushed apart to accommodate the inserted molecule. This structural alteration is usually accompanied by an increase in the relative specific viscosity of the DNA solution. In contrast, when binding occurs within the grooves of DNA, only slight increases or decreases in viscosity are generally observed. Gradual addition of each complex **C2** or **C4** (up to r = 1.0) to an 8 µM DNA solution resulted in a modest rise in the viscosity of CT-DNA (Figure S2, Supplementary Materials), though the effect was significantly less pronounced than that typically seen with classical intercalating agents [34].

#### 3.2.3. Fluorescence Quenching Measurements

**Figure 2:**
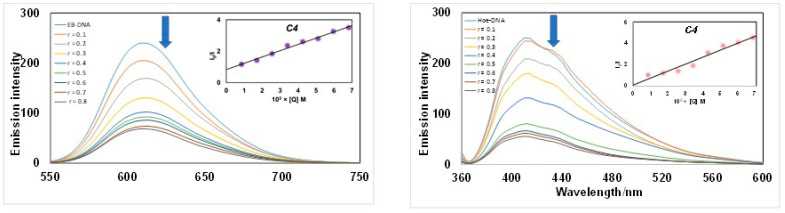
Emission spectra of EB/Hoe bound to DNA in the presence of **C4**. [EB] = 8.52 × 10^−5^ M; [Hoe] = 8.52 × 10^−5^ M; [DNA] = 8.52 × 10^−5^ M; [complex] = (0–6.82) × 10^−5^; λex = 527 nm; λex = 346 nm. Arrows show the intensity changes upon increasing the concentration of the complex. Inset graph: Plot of I_0_/I vs. [Q].

Fluorescence quenching experiments were conducted with DNA in the presence of EB (an intercalative binding indicator) for complexes **C1**–**C5**, and Hoe (a minor groove binding indicator) for complex **C4**. The results show that the gradual addition of increasing concentrations of the complexes to the EB-DNA or Hoe-DNA system solution led to a decrease in emission intensity (at 612 nm for EB and 410 nm for Hoe), indicating that the tested complexes are capable of displacing EB and Hoe (Figure 2 and Figure S3, Supplementary Materials).

The Stern–Volmer quenching constants (*K_s_*_v_) calculated for EB (Table 2), ranging from 10^3^–10^4^ M^−1^ for all complexes, reflect a moderate binding ability of these complexes toward DNA. The binding affinities of tested complexes follow the next order: **C5** > **C4** > **C3** > **C2** > **C1**, which is in agreement with the results obtained by Uv-Vis spectroscopy. Moreover, the comparison of *K*_sv_ values for complex **C4** in the presence of EB ((4.0 ± 0.1) × 10^4^ M^−1^) and Hoe ((6.6 ± 0.1) × 10^4^ M^−1^) indicates a preferential interaction of complex to DNA via minor groove binding over intercalative binding mode.

### 3.3. Molecular Docking Studies with DNA

Using molecular docking simulations, we focused on elucidating the binding modes, interaction strengths, and dominant binding sites on DNA. For this purpose, a selection of DNA macromolecules was made that covers both interaction modes of the complex. The best-docked conformations were analyzed, including parameters such as free energy of binding, key non-covalent interactions, beyond conventional hydrogen bonds, bridging electrostatic interactions, and hydrophobic contacts. The free energies of binding were then compared to a series of silver(I) complexes to interpret how structural and electronic features influence affinity for DNA. The calculated thermodynamic parameters for the best-docked conformations of tested compounds with DNA are displayed in Table 3. Negative values of the free energies of binding indicate that the mechanism of interaction of the tested complexes with DNA occurs spontaneously.

Intercalation was observed in the resulting docked poses with a 6 bp DNA fragment. This observation is consistent with the planar geometry of the silver(I) complexes; the aromatic moiety is inserted between the base pairs of the DNA double helix, causing structural changes and functional arrest. The results of the molecular docking are in agreement with the order of binding constants Kb from absorption spectroscopy studies and are **C1** < **C2** < **C3** < **C4** < **C5** for the investigated complexes. This binding pattern is based on the formation of π-stacking interactions, which are necessary for the stabilization of the complex formed with the DNA.

**Figure 3:**
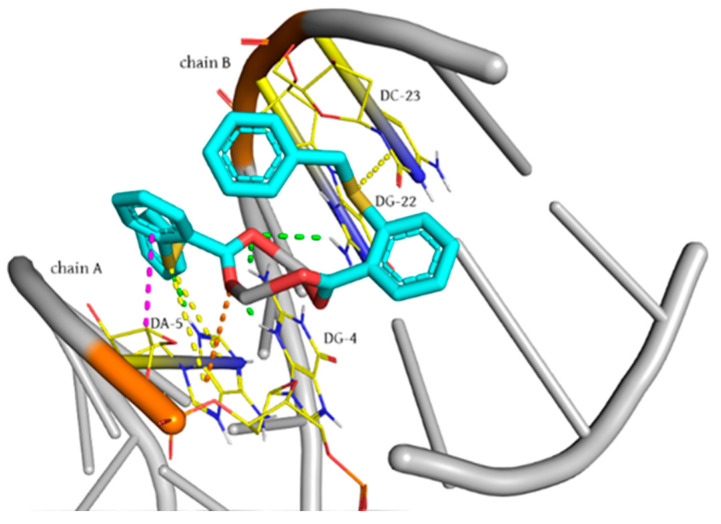
Molecular docked model of **C5** with DNA (PDB ID: 1BNA). The conventional hydrogen bonds are presented as green dotted lines, electrostatic interactions are depicted as orange dotted lines, the hydrophobic π-σ interaction is shown as a magenta dashed line, while π-S interactions are illustrated as yellow dotted lines.

Analysis of molecular docking data revealed that all complexes analyzed had very similar docking capabilities to the canonical B-DNA dodecamer; however, **C5** demonstrated the lowest free energy of binding, suggesting its superior binding potential. Compound **C5** behaves as a minor groove binder interacting with DNA. This type of complex binding is stabilized by the formation of key hydrogen bonds with A:DG4 (two bonds), A:DA5, and B:DG22 with interatomic distances of 1.83, 2.02, 2.26, and 2.98 Å, respectively. Additionally, important π-anion, π-sigma, and π-sulfur interactions with A:DA5 suggest a powerful affinity for **C5** for DNA grooves (Figure 3).

### 3.4. Albumin-Binding Studies

Serum albumin is the most abundant protein in blood plasma and plays an important role in the transport and delivery of many drugs to the sites of disease. Study of the interaction between biologically active compounds and proteins can give useful information about structural features that determine the therapeutic effectiveness of drugs, pharmacological response of drugs, and the design of dosage forms [49]. In many cases, these interactions are responsible for changes in the structure of proteins and can affect the biological properties of the original drug.

**Figure 4:**
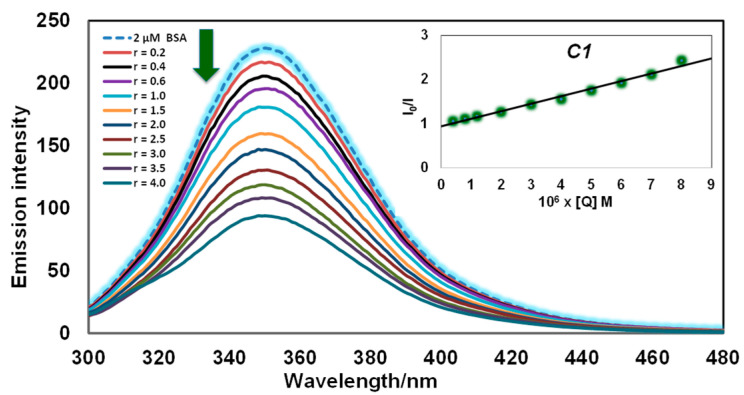
Emission spectra of BSA in the presence of **C1**. [BSA] = 2 μM, [complex] = 0–8 μM, λex = 295 nm. Arrows show the intensity changes upon increasing the concentration of the complex. Inset graph: Plot of I_0_/I vs. [Q].

This research focused on examining the binding interaction of BSA with the **C1**–**C5**. Upon excitation at 295 nm, BSA exhibits a prominent fluorescence emission peak centered at 352 nm [50]. Figure 4 and Figure S4 (Supplementary Materials) present the impact of compounds **C1**–**C5** on the emission spectra of BSA. Increasing the concentration of these compounds in the BSA solution resulted in a marked decrease in fluorescence emission at 352 nm. This reduction indicates a change in the tertiary structure of protein, most likely due to changes in the local environment of tryptophan residues upon complex binding to BSA [51].

The efficiency of BSA fluorescence quenching was assessed through the Stern–Volmer quenching constants (*K*_sv_) and the quenching rate constants (*k*_q_), as presented in Table 4. The results indicated that all examined complexes exhibited a strong quenching effect on BSA fluorescence. Moreover, the quenching rate constants ranging from 10^12^ to 10^13^ M^−1^ s^−1^ surpass those commonly associated with biopolymer fluorescence quenchers (around 2.0 × 10^10^ M^−1^ s^−1^), which supports the presence of a static quenching process [52,53].

Binding constants (K) and the number of binding sites (n) for the interactions between compounds **C1**–**C5** and BSA were determined using Scatchard plots (Figure S5, Supplementary Materials) based on the Scatchard equation (details in the experimental section). The comparison of the binding constants revealed that all complexes possess a strong binding affinity for BSA. The affinity of the complexes follows the decreasing order **C1** > **C2** > **C3** > **C4** > **C5**. Furthermore, the n values, which are approximately equal to 1, indicate that each complex binds to BSA in a 1:1 molar ratio.

The binding constants (*K*) of all examined complexes fall within the optimal range. Specifically, the *K* values are sufficiently high to promote efficient albumin binding and systemic transport yet remain below 10^15^ M^−1^—the association constant of avidin with various ligands, which is regarded as one of the strongest known non-covalent interactions. This ensures that, upon reaching target tissues, the complexes can be effectively released from albumin, thereby enabling their intended biological function [54].

### 3.5. Molecular Docking Studies with BSA

**Figure 5:**
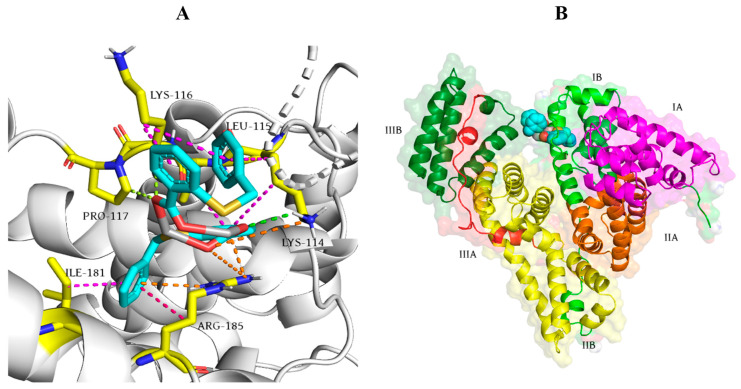
(**A**) Molecular docked model of **C5** with BSA (PDB ID: 4F5S). The conventional hydrogen bonds are presented as green dotted lines, C-H hydrogen bonds are shown as lemon-green dotted lines, electrostatic interactions are depicted as orange dotted lines, and hydrophobic interactions are shown as magenta dashed lines, including a single π-σ interaction presented as a hot-pink dotted line. (**B**) The position of **C5** (cyan, red, white, and yellow spheres) with regard to subdomains of BSA is presented.

Among the tested silver(I) complexes, **C5** demonstrated the lowest value of calculated free energy of binding (Table 5). The benzyl groups of this complex interacted with Lys114, Lys116, and Ile181 to form hydrophobic interactions of π-alkyl type. Further, the aromatic moiety of TSA established the same type of interactions with residue Lys114, Leu115, and Lys116, while the oxygen lone electron pair of the coordinate ligand achieved electrostatic interaction with the positively charged amino groups of Lys114 and Arg185. Additionally, the same amino acid residue formed a conventional hydrogen bond (2.68 and 2.95 Å) with the oxygen atom of the carbonyl group within the **C5**, while Lys116 and Pro117 were involved in the formation of a carbon–hydrogen bond (Figure 5). The other tested complexes showed lower binding affinity for BSA compared to **C5**, which is confirmed by the calculated thermodynamic parameters. The order of decreasing activity for the remaining complexes is in agreement with the experimentally obtained data. An exception from the experimental values is observed only in **C5**, which, due to the presence of an additional aromatic moiety, achieves a significantly higher number of hydrophobic interactions that contribute to a more favorable value of the free energy of binding. Established interactions with the BSA molecule indicate that the tested complexes are positioned within domain I, which is characterized by a dominantly α-helical structure. Ligand binding to this domain may induce conformational changes that affect the tertiary structure of the BSA and the position or environment of the Trp residue, in particular Trp134, which is located in subdomain IB. This finding was confirmed by emission spectra in which a decrease in the fluorescence intensity of BSA at 352 nm was observed after the addition of the investigated complexes to the BSA solution. Knowledge of the binding modes, binding constants, and dominant binding sites of the tested complexes with BSA enables the possibility of targeted action and drug delivery with a significant improvement in the safety profile.

### 3.6. In Vitro Cytotoxicity of Silver(I) Complexes with S-Alkyl Derivatives of Thiosalicylic Acid (***C1**–**C5***)

In order to validate cytotoxic capacity of newly synthesized silver(I) complexes with S-alkyl derivatives of thiosalicylic acid (**C1**–**C5**) and their ligands (**L1**–**L5**) in vitro, an MTT assay was performed. Cellular growth inhibition was tested on a panel of mouse and human cancer cell lines (breast cancer 4T1 and MDA-MB-468, colon cancer CT26 and HCT116, lung cancer LLC1 and A549) and non-cancer cell lines (mMSC and MRC-5) after an exposure period of 24 h and 48 h (Figures S6 and S7). Further, IC_50_ values were calculated and summarized in Table 6. The ligands, **L1**–**L5**, had minimal effects on cancer cell viability (IC_50_ > 208.38 µM), as illustrated in Figure S7. Interestingly, the lowest IC_50_ values were observed for **L5**. As shown in Figure S6, **C1**–**C5** complexes notably decreased the viability of all tested cell lines in a concentration-dependent manner. Unlike the control chemotherapeutic CDDP, time-dependent cytotoxic effects were absent for the tested silver(I) complexes, as most IC_50_ values after 48 h were similar to those after 24 h (Table 6). The **C1**–**C5** IC_50_ values were comparable to CDDP, with the exception of human and murine lung cancer cells, which were more sensitive to CDDP. Literature data confirm that silver complexes may demonstrate strong cytotoxic activity [18,21,22,23]. Rocha J. et al. reported remarkable cytotoxic activity of silver(I) complexes with phenanthroline ligand towards human ovarian carcinoma (A2780cis) and prostate cancer (DU-145) cell lines [23]. Also, distinct cytotoxic capacity was noticed for silver(I) complexes with triphenylphosphine and semicarbazone as ligands against human breast carcinoma cell lines (MDA-MB-231 and MCF-7) and prostate cancer (DU-145) [21]. Silver thiosulfate complex also reduced the viability of human breast carcinoma MCF-7 cells [22]. Silver(I) complexes bearing 1,10-phenanthroline and 2-formylpyridine thiosemicarbazones had noticeable cytotoxic capacity towards A549, MDA-MB-231, and MCF-7 cells [18]. Antiproliferative effects have been demonstrated for cooper(II) [55], palladium(II) [56], platinum(IV), and zinc(II) [57,58] complexes with S-alkyl derivatives of thiosalicylic acid.

In order to design drugs with low toxicity, it is important to evaluate cytotoxic effects on non-cancer cells. The cytotoxic capacity of **C1**–**C5** and **L1**–**L5** was also tested on non-cancer cell lines, murine mesenchymal stem cell line (mMSC), and human fibroblast cell line (MRC-5) (Table 6, Figures S6 and S7). Minimal cytotoxic capacity was observed for **L1**–**L5** compared to C–-**C5** (Table 6; Figure S7). After 24 h, **C1**–**C4** IC_50_ values for mMSC were 2–5 times lower compared to CDDP. By extending the incubation period, this difference disappeared (Table 6). The **C1**–**C5** IC_50_ values for MRC-5 were similar to CDDP after 24 h and higher after 48 h of treatment (Figure S6). Indexes of selectivity (IC_50_ mMSC or MRC-5/IC_50_ tumor cells) after 24 h and 48 h are presented in Table 7. The most pronounced cytotoxic selectivity was noticed for **C1**, **C2**, and **C3** relating to HCT116 cells. **C1**–**C3** complexes also had high SI towards CT26 cells. Optimal cytotoxic selectivity was spotted for **C3** complex toward 4T1 cells at both time points compared to CDDP (11.24 vs. 1.43 and 1.96 vs. 0.60, respectively, Table 7). The cytotoxic selectivity of silver complexes has also been confirmed in the literature [18,21,22,23]. Comparable or greater selectivity has been reported for Ag(I) complexes with heterocyclic thioamide ligands (SI ≈ 10) [59]. Water-soluble Ag–PTA–diclofenac coordination polymer achieved SI up to 28.1 [60]. Also, multiple Ag(I) N-heterocyclic carbene scaffolds commonly show SI > 1 across a panel of cancer cells [61,62]. Different silver complexes induce intrinsic apoptosis by raising ROS levels, blocking thioredoxin reductase, and damaging mitochondria [63,64,65]. Cancer cells are less tolerant to these changes than non-cancer cells, which might underlie the selective cytotoxicity of various silver complexes.

### 3.7. ***C3*** Reduces Tumor Growth In Vivo

**Figure 6:**
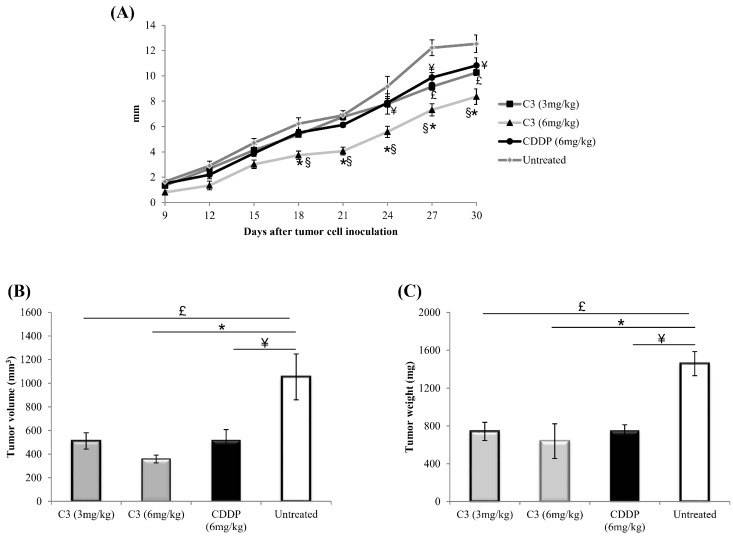
**C3** treatment inhibits breast cancer tumor growth. Tumor diameter (**A**), volume (**B**), and weight (**C**) in BALB/C mice i.p. treated with **C3** (3 mg/kg), **C3** (6 mg/kg), CDDP (6 mg/kg), or phosphate-buffered saline (untreated) (n = 8 animals per group). Data are presented as mean ± SEM. Statistical significance was determined by the Kruskal–Wallis test and Mann–Whitney U test as the post hoc test. (* *p* < 0.05 **C3** (6 mg/kg) vs. untreated; £ *p* < 0.05; **C3** (3 mg/kg) vs. untreated; § *p* < 0.05 **C3** (6 mg/kg) vs. CDDP (6 mg/kg); ¥ *p* < 0.05 CDDP (6 mg/kg) vs. untreated).

In order to further demonstrate the antitumor capacity of silver complexes in vivo, the murine 4T1 breast cancer model was selected. The 4T1 triple-negative breast cancer model is chosen because it remains a major cause of cancer mortality with pronounced chemoresistance. Although **C1**–**C3** complexes show a high cytotoxic capacity on 4T1 cells, **C3** was selected for further research due to the pronounced selectivity towards 4T1 cells compared to CDDP. After the orthotopic application of 4T1 cells into the mammary fat pad of BALB/C female mice and the appearance of a palpable tumor, mice were randomly assigned to four groups. BALB/C mice were treated with **C3** (3 mg/kg), **C3** (6 mg/kg), CDDP (6 mg/kg), or vehicle only (untreated group). After a short course treatment, on the 30th day after tumor inoculation, a significant reduction in breast cancer growth was observed in all pharmacologically treated animals compared to the untreated group (Figure 6A). As presented in Figure 6A, by day 18, there was no difference in tumor growth between pharmacologically treated animals and the untreated group. After that, tumor growth in **C3**-treated (6 kg/mg) animals, as well as **C3**-treated (3 mg/kg) and CDDP-treated (6 mg/kg) animals, was significantly slower compared to the untreated group. Tumor growth in the **C3**-treated group (3 mg/kg) was similar to that of the CDDP-treated group (6 mg/kg) during the entire experiment. Furthermore, at the end of the experiment, tumor volume and weight were significantly lower in all pharmacologically treated animals compared to the untreated group (Figure 6B,C). Our results are in line with previously published studies. Beta-D-glucose-reduced silver nanoparticles reduced 4T1 tumor growth and additionally remodeled the tumor microenvironment [66]. Similarly, albumin-coated silver nanoparticles reduced breast cancer (4T1) tumor growth in vivo [67].

**Figure 7:**
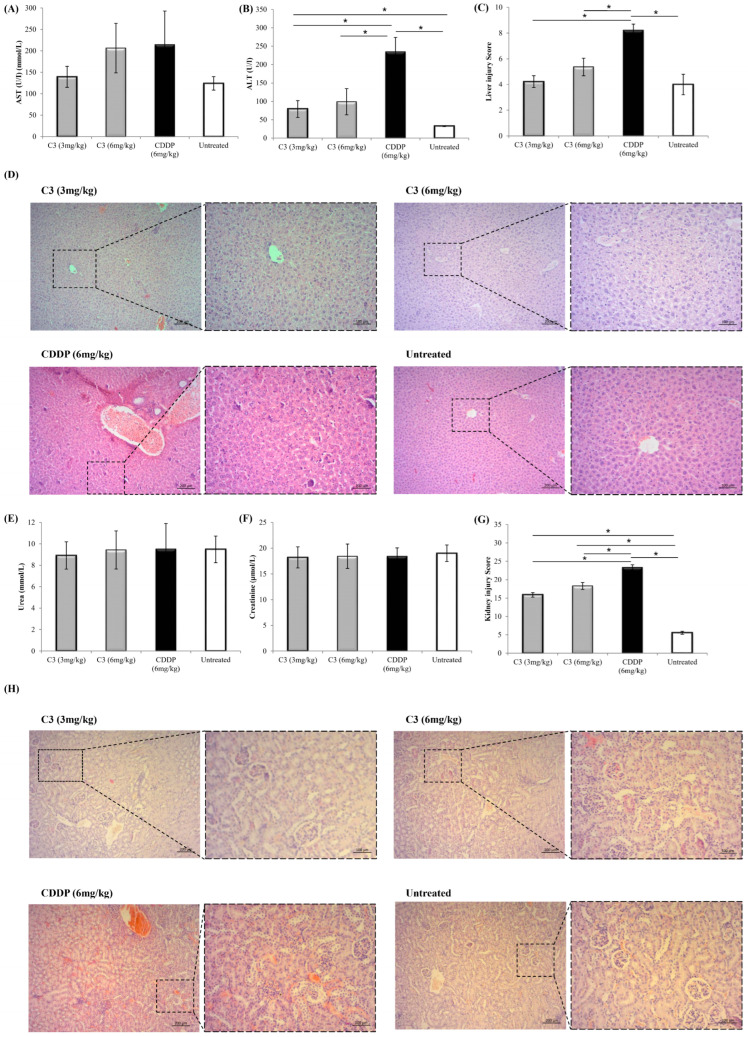
Safety profile after **C3** treatment. Serum concentration of AST (**A**), ALT (**B**), total histological score of liver injury (**C**), representative hematoxylin and eosin staining of liver tissue sections (magnification at ×100 and ×200) (**D**), urea (**E**), creatinine (**F**), total histological score of kidney injury (**G**), and representative hematoxylin and eosin staining of kidney tissue sections (magnification at ×100 and ×200) (**H**) of tumor-bearing BALB/C mice after treatment with **C3** (3 mg/kg), **C3** (6 mg/kg), CDDP (6 mg/kg), or phosphate-buffered saline (untreated). Data are presented as mean ± SD. Statistical significance was determined by the Kruskal–Wallis test and Mann–Whitney U test as a post hoc test. * *p* < 0.05.

It is known that the main limitation of CDDP, as the pioneer of metal-based drugs, is the high systemic toxicity. The cornerstone of developing new chemotherapeutics is to synthesize a drug with the same clinical efficacy and a better safety profile. As all animals survived until the end of the experiment, the next goal was to determine **C3**-induced toxicity in vivo through biochemical and histopathological analyses. On the 30th day after 4T1 cell inoculation, the serum levels of transaminases (ALT and AST), urea, and creatinine were measured, and histopathological properties of the target organs, liver and kidneys, were evaluated. As presented in Figure 7A, no differences in AST levels were observed among the groups. Elevated serum levels of ALT were detected in all pharmacologically treated animals compared to untreated animals (Figure 7B). Histopathological evaluation of H&E-stained liver samples did not reveal a significant difference in liver injury histological score (Knodel score) between **C3**-treated and untreated animals (Figure 7C). CDDP treatment led to liver changes like a piecemeal necrosis, focal lytic necrosis, and portal inflammation (Figure 7D). Further, no differences in the serum levels of urea and creatinine were observed among all tested groups (Figure 7E,F). All pharmacologically treated animals had significantly higher values of kidney injury scores (Figure 7G). Hypercellularity of glomeruli, focal tubular basophilia, interstitial mononuclear leukocyte infiltration, and atrophic and dilated tubules were observed in untreated breast cancer-bearing animals (Figure 7H). Additionally, hypertrophy of parietal epithelial cells, dilatation of tubules, and tubular cell necrosis and segmental atrophy of tubular loss were noticed in the **C3**-treated groups and the CDDP-treated group. Kidney injury scores were significantly lower in **C3**-treated groups compared to CDDP-treated animals. These findings suggested that **C3** might be as effective as CDDP and less toxic in vivo. Beyond hepatic and renal safety, it has been shown that silver-based compounds might have other potential systemic effects. Silver particulates can accumulate in the spleen, lymph nodes, lungs, heart, and brain [68,69]. Future studies should evaluate pharmacokinetics, biodistribution, and safety to clarify potential limits in clinical translation.

### 3.8. ***C3*** Induces Apoptotic Cell Death and Interferes with Mitochondrial Function

**Figure 8:**
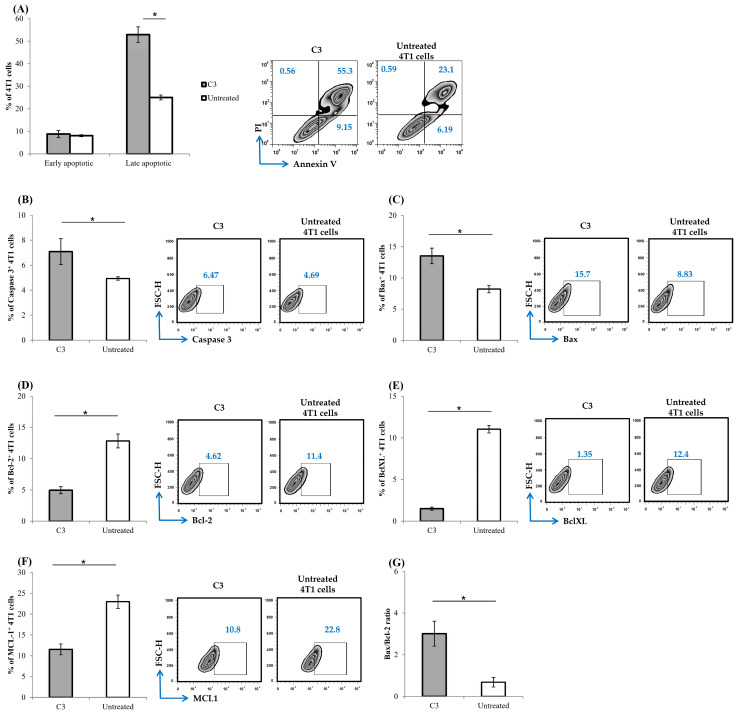
4T1 cells undergo apoptosis after **C3** treatment. Apoptotic rates of **C3**- treated (0.5 μM for 24 h) as well as untreated 4T1 cells, were determined by flow cytometry using Annexin V (FITC) and PI double staining (**A**). Analysis of casapse-3 (**B**), Bax (**C**), Bcl-2 (**D**), BclXL (**E**), and Mcl-1 (**F**) expression in 4T1 cells exposed to **C3** (0.5 μM for 24 h) using flow cytometry, and the Bax/Bcl-2 ratio was calculated (**G**). Representative dot plots are presented. The data are shown as averages ± SD of a 3 independent experiments. Mann-WhitneyMann–Whitney U test: * *p* < 0.05 compared with the untreated group.

After the antitumor potential of the **C3** complex has been proven, the next goal of the study is to determine the underlying mechanism of action. In the following apoptosis/necrosis measurement, 4T1 cells had been treated with 0.5 µM of **C3** for 24 h, stained with Annexin V (AnnV) and propidium iodide (PI), and after that, apoptotic/necrotic rates were determined by flow cytometry (Figure 8A). After **C3** treatment, the majority of 4T1 cells were detected as AnnV^+^/PI^+^, indicating that **C3** treatment increased the percentage of late apoptotic 4T1 cells (Figure 8A). No difference was observed in the percentage of early apoptotic cells between the **C3**-treated and untreated groups (Figure 8A). Similar results were observed for **C2** (Figure S8). The results of other studies indicate that various silver(I) complexes could induce apoptosis in cancer cells [18,70]. As these results suggested that 4T1 cells were more prone to apoptosis after **C3** treatment, we further investigated the possible apoptotic pathway. Caspase-3, known as the executioner caspase, plays a key role in apoptotic cascades [20]. The expression of cleaved caspase-3 was significantly increased in **C3**-treated compared to untreated 4T1 cells (Figure 8B). In addition, as programmed cell death is regulated by the balance of pro- and anti-apoptotic molecules [71], expression of Bax, Bcl-2, Bcl-XL, and MCL-1 in 4T1 cells following **C3** treatment was conducted (Figure 8C–F). The expression of pro-apoptotic Bax in 4T1 cells was increased (Figure 8C), while percentages of Bcl-2^+^ (Figure 8D), Bcl-XL^+^ (Figure 8E), and MCL-1^+^ (Figure 8F) 4T1 cells were significantly decreased after **C3** treatment compared to untreated 4T1 cells. The Bax/Bcl-2 ratio, an important sign of a cell’s ability to undergo apoptosis, was higher after **C3** treatment compared to untreated 4T1 cells (Figure 8G), suggesting the predominance of pro-apoptotic molecules and a subsequent increased possibility of apoptosis. The results demonstrated that the apoptosis was induced after **C3** treatment. It has been reported that various silver(I) complexes trigger apoptosis via mitochondrial disruption and caspase activation [72,73,74,75]. Based on the fact that loss of mitochondrial membrane potential (MMP) is a crucial event in the early stage of cell death [76], we further stained **C3**-treated and untreated 4T1 cells with Rhodamine-123 for membrane potential analysis.

**Figure 9:**
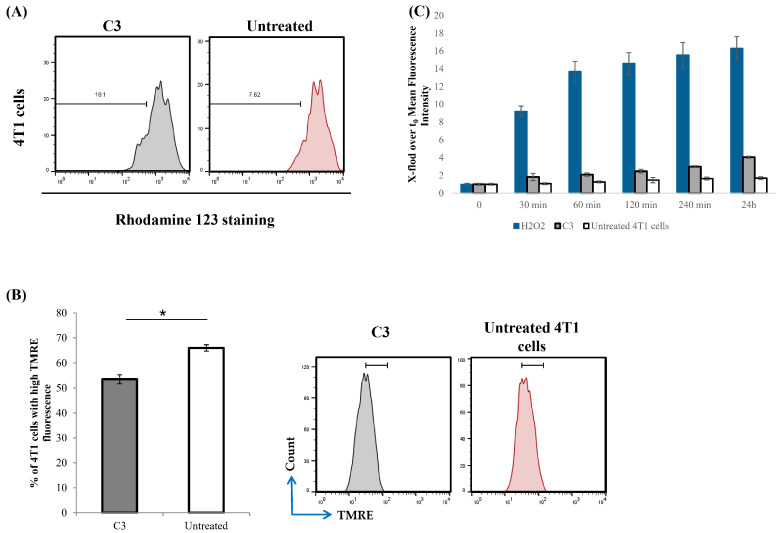
**C3** treatment modulates mitochondrial function. The alteration of mitochondrial transmembrane potential following **C3** (0.5 μM) was assessed via flow cytometry utilizing the fluorescent dye Rhodamine-123. The percentage of Rhodamine-123-negative cells was shown (**A**). 4T1 cells following **C3** treatment (0.5 μM) were stained with TMRE for determining mitochondrial membrane potential (**B**). The data are shown as averages ± SD of 3 independent experiments. Mann–Whitney U test: * *p* < 0.05 compared with the untreated group. Representative dot plots are presented. DCFDA dye was used to stain H_2_0_2_-treated (positive control), **C3**-treated, and untreated 4T1 cells. In order to determine the relative amount of cellular ROS, flow cytometry was used. The results are presented as fold change of mean fluorescence intensity at 0 min (**C**). The data are shown as averages ± SD of 3 independent experiments.

**C3** treatment increased the percentage of Rhodamine-123 negative 4T1 cells, indicating that **C3** might reduce mitochondrial transmembrane potential (Figure 9A). As shown in Figure 9B, incubation of 4T1 cells in the presence of **C3** (0.5 µM) led to a significant decrease in TMRE fluorescence, indicating the loss of mitochondrial membrane potential. As mitochondria are considered the primary source of ROS and overproduction of ROS is linked to apoptosis [77], we examined changes in **C3**-mediated ROS production. **C3**-treated (0.5 µM) and nontreated 4T1 cells were stained with 2′,7′-dichlorodihydrofluorescein diacetate (H_2_DCFDA), analyzed by flow cytometry, and quantitative data are presented in Figure 9C. The **C3** complex increased the level of intracellular ROS after a 24 h incubation, suggesting ROS as a stimulator of apoptotic death. Taken together, **C3** may promote apoptosis by inducing ROS production, reducing mitochondrial membrane potential, activating caspase-3, increasing pro-apoptotic, and decreasing anti-apoptotic markers. Other studies have confirmed that silver(I) complexes favor the intrinsic pathway of apoptosis [18,22,72,74]. Mitochondrial depolarization, cytochrome-c release, and activation of caspase-9/-3 were noticed for phosphine-based Ag(I) systems and mitochondrial targeting for Ag(I) N-heterocyclic carbenes [63,78]. Like **C3**, silver agents also increased the pro-apoptotic Bax and/or decreased the anti-apoptotic Bcl-2, which makes tumor cells more likely to undergo apoptosis [72,79].

### 3.9. ***C3*** Suppresses Breast Cancer Cell Proliferation

**Figure 10:**
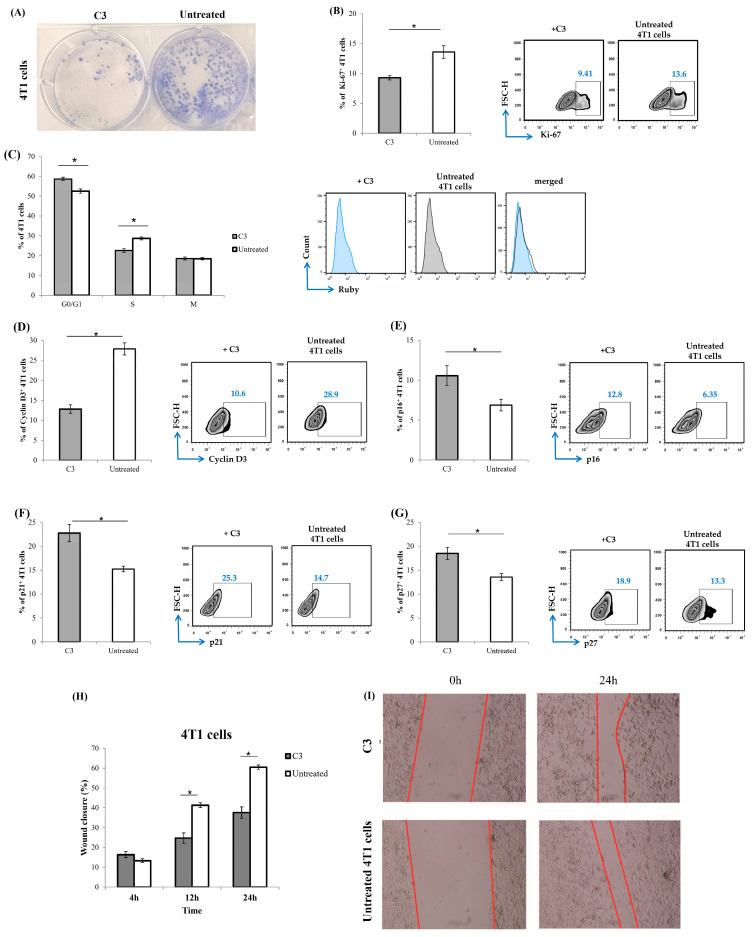
**C3** promotes cell cycle arrest in 4T1 cells at the G0/G1 checkpoint. The image shows colony formation of 4T1 cells after treatment with **C3** at 0.5 μM for 14 days (**A**). Analysis of Ki-67 expression in 4T1 cells after **C3** treatment (0.5 μM for 24 h), as well as untreated cells, was conducted by flow cytometry (**B**). 4T1 cells were treated with **C3** (0.5 μM for 24 h) or untreated and stained with Vybrant^®^ DyeCycle^TM^ Ruby stain in order to examine cell cycle progression using flow cytometry. Results are presented as the percentage of cells in various phases of the cell cycle (**C**). Analysis of cyclin D3 (**D**), p16 (**E**), p21 (**F**), and p27 (**G**) expression following **C3** treatment using flow cytometry. Representative dot plots are presented. The data are shown as averages ± SD of 3 independent experiments. Mann–Whitney U test: * *p* < 0.05 compared with the untreated group. Impact of **C3** (0.5 μM) on 4T1 tumor cell migration assessed via the scratch assay. Wound healing assay and quantification of migration area in the 4T1 cells treated with **C3** or untreated cells after 4 h, 12 h, and 24 h (**H**,**I**). Data were expressed as mean ± SD in more than 3 microscopic fields. Mann–Whitney U test: * *p* < 0.05 compared with the untreated group.

Apoptosis and the cell cycle are linked processes. Many signals that trigger apoptosis may also affect the cell cycle, and vice versa [80]. Antitumor agents may inhibit cellular proliferation by directly targeting DNA, disrupting the cell cycle, or inhibiting growth signals within the cell [81]. Excessive concentration of ROS may overcome the capacity of cellular antioxidant defenses and affect the cell cycle and cell proliferation [82]. To further investigate the anti-proliferative effect of **C3**, a colony formation assay was performed. 4T1 cells had been incubated in the presence of **C3** (0.5 µM) for 14 days. **C3** induced an almost total inhibition of 4T1 colony formation, suggesting that **C3** inhibited long-term cellular proliferation (Figure 10A). Ki-67 is a key marker for identifying proliferating cells as it is expressed during all cell cycle stages [83]. The percentage of Ki-67^+^ 4T1 cells after **C3** treatment was significantly lower compared to untreated 4T1 cells (Figure 10B). To elucidate the mechanisms of the reduction of 4T1 cell proliferation after **C3** treatment, further analyses were focused on cell cycle regulation. The cell cycle profile of 4T1 cells was determined by flow cytometry after treatment with **C3** (0.5 µM) for 24 h. As presented in Figure 10C, **C3** significantly increased the percentage of 4T1 cells in G0/G1 phase and simultaneously decreased the percentage of 4T1 cells in S phase. Cell cycle is tightly regulated by the simultaneous activity of cyclins, cyclin-dependent kinases (CDK), and inhibitors of CDK. Type D cyclins are crucial for switching the cell cycle from G1 to S phase [81]. The major regulator of the G1 phase is the cyclin D-CDK4/6 complex. This complex is negatively regulated by the inhibitors of CDK, such as INK4 family (p16, p15, p18, and p19), while KIP/CIP family inhibitors of CDK (p21, p27, and p57) may facilitate stabilization of this complex and inhibit cyclin E–CDK2 complex [81,84]. Following **C3** treatment, the percentage of cyclin D3^+^ 4T1 cells was significantly lower (Figure 10D), while percentages of p16^+^ 4T1 cells, p21^+^ 4T1 cells, and p27^+^ 4T1 cells (Figure 10E–G) were elevated compared to untreated 4T1 cells. Presented data suggest that **C3** inhibits 4T1 cell proliferation by inducing cell cycle arrest in the G0/G1 phase via upregulation of p16, p21, and p27 expression and downregulation of cyclin D3 expression. Disruption of the cancer cell cycle was noticed after treatment with various silver(I) complexes [72]. Increased percentage of human breast cancer cells in the sub-G1 phase was recorded after treatment with silver(I) complex containing 2- 1,10-phenanthroline formylpyridine and thiosemicarbazones [18]. Treatment with silver thiosulfate complex led to cell cycle arrest at the G1 phase [12], as well as silver(I) dipeptide complexes [85]. Human breast cancer cell cycle arrest at G2/M phase was detected after silver(I) dibenzoylmethane-based complexes treatment [73].

A hallmark of elevated metastatic potential in cancer cells is cell migration [86]. Scratch assay was performed in order to determine whether **C3** (0.5 µM) treatment can affect 4T1 cell migration. Quantification of the wound closure was conducted using ImageJ software (version 1.54.0 NIH, USA). As presented in Figure 10H, **C3** treatment significantly reduced the migration ability of 4T1 cells after 12 and 24 h compared to untreated cells. The results demonstrate that **C3** treatment markedly diminished the proliferation ability of murine breast cancer cells, which may consequently affect the development of metastatic cancer.

Analysis of the structural features and biological results reveals a clear correlation between the structures of the silver complexes (**C1**–**C5**) and their cytotoxic activity. The bidentate coordination of ligands plays a crucial role in stabilizing the newly formed complexes, altering electron distribution, and enhancing biological activity compared to the free ligands. The varying lengths of the alkyl chains in the ligands are a key factor influencing their antitumor potential. Complexes **C1**–**C3**, characterized by simpler structures and shorter alkyl chains, exhibit moderate cytotoxicity but demonstrate greater selectivity toward tumor cells compared to normal ones. In contrast, complex **C5**, which possesses the highest degree of lipophilicity and the most voluminous structure, exhibits the lowest IC_50_ values against several tumor cell lines (particularly HCT116 and CT26). This suggests that increased lipophilicity facilitates membrane permeation and stronger interactions with biomolecules. However, this effect is accompanied by reduced selectivity, as **C5** also shows considerable toxicity toward normal cells (MRC-5). Based on the obtained in vitro results, complex **C3** displays the most favorable balance between activity and selectivity and was therefore selected for subsequent in vivo studies.

## 4. Conclusions

**Figure 11:**
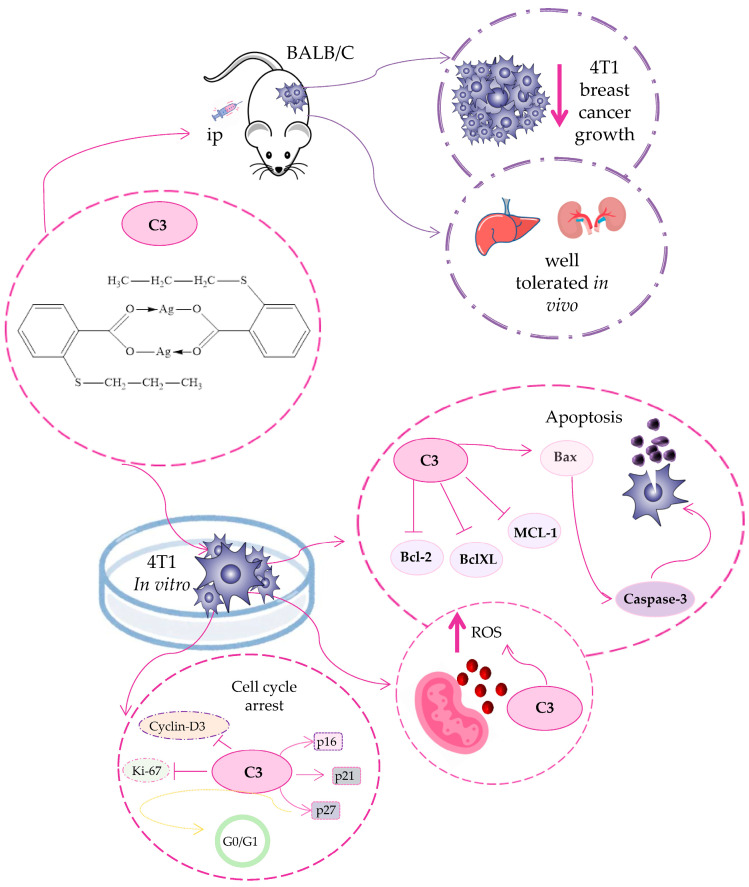
**C3** mechanism of action.

In summary, the results of the studied interactions of **C1**–**C5** with DNA/BSA revealed moderate to high binding affinity to macromolecules. The quenching constant values (Ksv) observed in BSA binding studies indicate that the compounds interact with serum albumin to enable effective transport and prolonged presence in the bloodstream. At the same time, these interactions are not so strong as to prevent the compounds from being released upon reaching their target tissues. The moderate Ksv values observed for DNA indicate that interactions are not excessively strong, which minimizes the risk of permanent DNA damage and possible side effects. This controlled interaction profile supports the compounds’ potential efficacy and safety in vivo. Silver(I) complexes with S-alkyl derivatives of thiosalicylic acid reduced the viability of various cancer cells in a concentration-dependent manner. **C3** demonstrated a significant antitumor impact both in vitro and in vivo. Systemic administration of **C3** over a short course interval was well tolerated. Potential tumoricidal capabilities of **C3** include the induction of apoptosis in cancer cells through a complex interplay of Bcl-2 family proteins and ROS, followed by inhibition of cellular proliferation (Figure 11). The tumoricidal and antiproliferative capacities of silver(I) complexes with S-alkyl derivatives of thiosalicylic acid may represent a promising candidate for the development of new anticancer drugs. Future studies are necessary to optimize the administration route, gain insight into pharmacokinetics and biodistribution, assess metastatic inhibition in vivo, and find structural modifications to improve selectivity or reduce toxicity.

## Figures and Tables

**Table 1:** Infrared data of the carboxyl group for newly synthesized silver(I) complexes.

Compound	Carboxylate Group		
	ν_asym_ COO^−^	ν_sym_ COO^−^	∆*ν*
**C1**	1537 cm^−1^	1369 cm^−1^	168
**C2**	1537 cm^−1^	1370 cm^−1^	167
**C3**	1547 cm^−1^	1366 cm^−1^	181
**C4**	1548 cm^−1^	1368 cm^−1^	180
**C5**	1550 cm^−1^	1377 cm^−1^	173

**Table 2:** CT-DNA-binding constants (*K*_b_), Gibbs free energies (Δ*G*), and Stern–Volmer constants (*K*_sv_) of complexes **C1**–**C5**.

Compound	*K*_b_ (M^−1^)	Δ*G* (KJ mol^−1^)	*K*_sv_ EB (M^−1^)
**C1**	(7.5 ± 0.1) × 10^4^	−27.8	(6.0 ± 0.1) × 10^3^
**C2**	(9.1 ± 0.1) × 10^4^	−28.3	(1.1 ± 0.1) × 10^4^
**C3**	(2.2 ± 0.1) × 10^5^	−30.5	(1.4 ± 0.1) × 10^4^
**C4**	(3.5 ± 0.1) × 10^5^	−31.6	(4.0 ± 0.1) × 10^4^
**C5**	(6.6 ± 0.2) × 10^5^	−33.2	(6.8 ± 0.1) × 10^4^

*K*_b_—CT-DNA-binding constant; Δ*G*—Gibbs free energies; *K*_sv_—Stern–Volmer constant.

**Table 3:** The calculated thermodynamic parameters for the best-docked conformations of silver(I) complexes with DNA.

PDB ID	Complex	ΔG (kcal/mol)	ΔG_vdw + hbond + desolv_ (kcal/mol)	ΔG_elec_ (kcal/mol)	ΔG_Intermol. Energy_ (kcal/mol)	ΔG_total_ (kcal/mol)	ΔG_tor_ (kcal/mol)	ΔG_unb_ (kcal/mol)
1Z3F	**C1**	−6.79	−7.72	−0.17	−7.89	−0.57	1.10	−0.57
	**C2**	−6.50	−8.09	−0.05	−8.15	−0.82	1.65	−0.82
	**C3**	−6.23	−8.39	−0.03	−8.42	−0.98	2.20	−0.98
	**C4**	−5.95	−8.55	−0.14	−8.69	−1.51	2.74	−1.51
	**C5**	−7.62	−9.89	0.07	−9.81	−2.23	2.20	−2.23
1BNA	**C1**	−8.18	−10.88	−0.04	−10.92	−1.50	2.74	−1.50
	**C2**	−8.30	−10.38	−0.11	−10.49	−2.09	2.20	−2.09
	**C3**	−8.50	−10.56	−0.13	−10.69	−0.88	2.20	−0.88
	**C4**	−9.07	−10.14	−0.04	−10.17	−0.56	1.10	−0.56
	**C5**	−9.28	−10.88	−0.04	−10.92	−0.69	1.65	−0.69

**Table 4:** The BSA binding constants and parameters (*K*_sv_, *k*_q_, *K*, *n*) derived for **C1**–**C5** complexes.

Compound	*K*_sv_ (M^−1^)	*k*_q_ (M^−1^ s^−1^)	*K* (M^−1^)	*n*
**C5**	(5.8 ± 0.1) × 10^4^	(5.8 ± 0.1) × 10^12^	(8.3 ± 0.1) × 10^4^	0.8
**C4**	(6.3 ± 0.1) × 10^4^	(6.3 ± 0.1) × 10^12^	(8.9 ± 0.1) × 10^4^	0.8
**C3**	(9.6 ± 0.1) × 10^4^	(9.6 ± 0.1) × 10^12^	(1.1 ± 0.1) × 10^5^	1.1
**C2**	(1.4 ± 0.1) × 10^5^	(1.4 ± 0.1) × 10^13^	(1.3 ± 0.1) × 10^5^	1.1
**C1**	(1.7 ± 0.1) × 10^5^	(1.7 ± 0.1) × 10^13^	(1.7 ± 0.1) × 10^5^	0.8

*K*_sv_—Stern–Volmer constant; *k*_q_—quenching rate constant; *K*—binding constant; *n*—number of binding sites.

**Table 5:** The calculated thermodynamic parameters for the best-docked conformations of silver(I) complexes with BSA.

PDB ID	Complex	ΔG (kcal/mol)	ΔG_vdw + hbond + desolv_ (kcal/mol)	ΔG_elec_ (kcal/mol)	ΔG_Intermol. Energy_ (kcal/mol)	ΔG_total_ (kcal/mol)	ΔG_tor_ (kcal/mol)	ΔG_unb_ (kcal/mol)
	**C1**	−8.55	−10.73	−0.01	−10.74	−1.13	2.20	−1.13
	**C2**	−8.07	−9.18	0.01	−9.17	−0.73	1.10	−0.73
4F5S	**C3**	−8.02	−9.71	0.04	−9.67	−1.06	1.65	−1.06
	**C4**	−7.94	−10.72	0.03	−10.69	−0.97	2.74	−0.97
	**C5**	−8.87	−11.12	0.05	−11.07	−2.50	2.20	−2.50

**Table 6:** **C1**–**C5** IC_50_ values determined by MTT assay. Data are presented as average ± S.D for three independent experiments.

Cell Line	Time	**C1**	**C2**	**C3**	**C4**	**C5**	**L1**	**L2**	**L3**	**L4**	**L5**	CDDP
4T1	24 h	0.33 ± 0.21	0.57 ± 0.15	0.25 ± 0.19	0.64 ± 0.28	18.65 ± 1.15	1420.66 ± 68.58	628.32 ± 40.51	954.56 ± 63.34	406.83 ± 35.1	290.96 ± 20.47	11.98 ± 1.98
	48 h	4.00 ± 0.95	4.84 ± 0.92	2.13 ± 1.17	4.55 ± 0.88	4.20 ± 1.48	442.84 ± 30.52	499.79 ± 7.60	499.69 ± 30.48	416.85 ± 61.42	375.12 ± 40.37	5.17 ± 0.98
MDA-MB-468	24 h	4.19 ± 0.98	8.67 ± 1.13	15.47 ± 1.21	15.06 ± 1.56	18.77 ± 0.69	885.89 ± 35.22	721.63 ± 55.32	612.78 ± 49.57	899.17 ± 29.55	457,12 ± 45.99	4.06 ± 0.97
	48 h	4.37 ± 0.41	5.43 ± 0.34	8.76 ± 1.24	9.88 ± 0.962	8.60 ± 1.65	511.35 ± 74.55	767.78 ± 28.84	528.51 ± 51.96	407.51 ± 12.74	337.18 ± 18.23	3.47 ± 0.47
CT26	24 h	2.03 ± 0.59	1.08 ± 0.03	1.58 ± 0.82	7.71 ± 0.21	29.94 ± 2.12	664.54 ± 28.84	708.81 ± 83.91	533.77 ± 27.72	647.14 ± 11.14	389.16 ± 9.59	16.94 ± 1.42
	48 h	1.24 ± 0.47	3.21 ± 0.39	3.29 ± 1.28	4.45 ± 0.37	7.19 ± 0.38	701.74 ± 61.08	464.56 ± 39.52	683.57 ± 61.21	367.83 ± 58.31	369.88 ± 18.69	1.29 ± 0.11
HCT 116	24 h	3.24 ± 0.67	3.01 ± 1.31	1.19 ± 0.21	16.73 ± 2.79	44.45 ± 3.68	736.60 ± 60.02	713.96 ± 30.01	649.43 ± 46.58	669.11 ± 43.11	246.99 ± 19.02	63.31 ± 3.21
	48 h	1.24 ± 0.065	1.04 ± 0.06	1.46 ± 0.83	11.28 ± 2.13	3.96 ± 0.97	1341.94 ± 136.14	1161.57 ± 98.7	1335.21 ± 54.16	951.03 ± 32.64	208.38 ± 28.84	15.78 ± 1.28
LLC1	24 h	39.75 ± 1.07	18.25 ± 0.93	41.58 ± 1.27	57.77 ± 1.64	29.09 ± 0.90	590.55 ± 47.85	566.5 ± 17.67	805.5 ± 31.66	1489.10 ± 71.82	741.99 ± 37.81	21.82 ± 1.11
	48 h	32.02 ± 1.47	20.21 ± 0.79	20.31 ± 1.18	46.53 ± 1.32	24.23 ± 0.78	947.42 ± 41.37	960.29 ± 63.64	681.48 ± 32.23	670.09 ± 48.13	860.98 ± 60.96	3.51 ± 0.92
A549	24 h	58.27 ± 1.91	37.75 ± 1.01	55.77 ± 1.83	48.61 ± 1.92	24.35 ± 1.57	1059.61 ± 75.52	723.31 ± 37.99	1414.53 ± 75.31	494.87 ± 3.58	394.57 ± 39.06	23.22 ± 1.77
	48 h	48.62 ± 1.057	50.23 ± 1.074	66.27 ± 1.18	99.03 ± 3.85	59.92 ± 2.02	1529.22 ± 82.21	540.11 ± 34.32	499.94 ± 47.29	489.72 ± 22.01	643.56 ± 26.17	14.00 ± 1.44
mMSC	24 h	2.84 ± 0.21	4.29 ± 0.92	2.81 ± 0.5	9.41 ± 1.13	28.65 ± 1.02	815.29 ± 36.59	1203.02 ± 67.98	843.33 ± 31.09	540.67 ± 9.85	434.82 ± 10.09	17.14 ± 1.15
	48 h	2.54 ± 0.26	4.52 ± 0.48	4.22 ± 0.33	1.67 ± 0.27	2.16 ± 0.62	697.5 ± 7.78	843.33 ± 31.09	1139.57 ± 41.61	1884.32 ± 96.77	636.60 ± 41.88	3.11 ± 0.93
MRC-5	24 h	46.3 ± 1.52	38.04 ± 1.91	38.1 ± 1.13	26.49 ± 0.91	46.04 ± 1.37	658.33 ± 53.23	538.45 ± 20.63	637.81 ± 50.98	947.93 ± 50.03	331.54 ± 29.02	40.5 ± 0.57
	48 h	53.25 ± 1.27	43.17 ± 1.23	43.34 ± 1.1	15.34 ± 0.73	46.65 ± 1.87	487.71 ± 41.56	384.10 ± 20.53	628.26 ± 60.49	718.50 ± 39.71	447.87 ± 36.04	27.35 ± 0.72

**Table 7:** Index of selectivity (IC_50_ noncancer cells/ IC_50_ tumor cells) of **C1**–**C5**.

	Selectivity Index (IC_50_ mMSC or MRC-5/ IC_50_ Cancer Cell)						
Cell Line	Time	**C1**	**C2**	**C3**	**C4**	**C5**	CDDP
4T1	24 h	8.60	7.53	11.24	14.70	1.549	1.43
	48 h	0.64	0.93	1.98	0.37	0.51	0.60
CT26	24 h	1.40	3.97	1.78	1.22	0.96	1.01
	48 h	2.04	1.41	1.28	0.38	0.30	2.41
LLC1	24 h	0.07	0.24	0.07	0.16	0.98	0.79
	48 h	0.08	0.22	0.21	0.04	0.09	0.89
MDA-MB-468	24 h	11.05	4.39	2.46	1.76	2.45	9.98
	48 h	12.18	7.95	4.95	1.55	5.42	7.88
HCT-116	24 h	14.29	12.64	32.02	1.58	1.04	0.64
	48 h	42.94	41.51	29.68	1.36	11.78	1.73
A549	24 h	0.79	1.01	0.68	0.55	1.89	1.74
	48 h	1.10	0.86	0.65	0.15	0.78	1.95

## Data Availability

The raw data supporting the conclusions of this article would be available by the authors on request.

## Supplementary Materials

The following supporting information can be downloaded at https://www.mdpi.com/article/10.3390/pharmaceutics17101340/s1. NMR and FTIR spectra of free ligands and complexes. Figure S1. Absorption spectra of **C2**–**C5** at 25 °C in PBS buffer upon addition of CT-DNA. [complex] = 8 × 10^−6^ M; [DNA] = (0–6.4) × 10^−6^ M. The arrow shows the change in the absorbance with the increase in DNA concentration. Inset: Plot of [DNA]/(*ε*_A_* − ε*_f_) vs. [DNA]. Figure S2. Relative viscosity (η/η_0_) ^1/3^ of CT-DNA (8 µM) in PBS buffer solution with the addition of increasing amounts (r) of **C2** and **C4** complexes. Figure S3. Emission spectra of EB bound to DNA in the presence of **C1**, **C2**, **C3**, and **C5**. [EB] = 8.52 × 10^−5^ M; [DNA] = 8.52 × 10^−5^ M; [complex] = (0–6.82) × 10^−5^; λex = 527 nm. Arrows show the intensity changes upon increasing the concentration of the complex. Inset graph: Plot of *I*_0_/*I* vs. [Q]. Figure S4. Emission spectra of BSA in the presence of **C2**–**C5**. [BSA] = 2 μM; [complex] = 0–8 μM; λex = 295 nm. Arrows show the intensity changes upon increasing the concentration of the complex. Inset graph: Plot of *I*_0_/*I* vs. [Q]. Figure S5. Scatchard plots for **C1**–**C5** complexes. Figure S6. Dose-dependent cytotoxicity of silver(I) complexes with S-alkyl derivatives of thiosalicylic acid. The effect of **C1**–**C5** on the viability of 4T1 (A), MDA-MB-468(B), CT26(C), HCT116(D), LLC1(E), A549(F), mMSC(G), and MRC-5(H) cells after a period of incubation of 24 h and 48 h, analyzed with the MTT assay. All data are presented as mean values ± SD from three independent experiments performed in triplicate. Figure S7. Dose-dependent cytotoxicity of S-alkyl derivatives of thiosalicylic acid. The effect of **L1**–**L5** on the viability of 4T1 (A), MDA-MB-468(B), CT26(C), HCT116(D), LLC1(E), A549(F), mMSC(G), and MRC-5(H) cells after a period of incubation of 24 h and 48 h, analyzed with the MTT assay. All data are presented as mean values ± SD from three independent experiments performed in triplicate. Figure S8. CT26 and HCT116 cells underwent apoptosis after **C2** treatment. Apoptotic rates of **C2**-treated (0.5 μM for 24 h) as well as untreated CT26 and HCT116 cells were determined by flow cytometry using Annexin V (FITC) and PI double staining. The data are shown as averages ± SD of 3 independent experiments. Mann–Whitney U test: * *p* < 0.05 compared with the untreated group. Figure S9. Relationship between DNA-binding constants (Kb) and IC_50_ values.
