# Supplementary material for: Silver(I) Complexes Bearing S-Alkyl Thiosalicylic Acid Derivatives: DNA/BSA Binding and Antitumor Activity In Vitro and In Vivo
Source: Pharmaceutics. 2025 Oct 16;17(10):1340. doi: 10.3390/pharmaceutics17101340 (PMC12567082; doi:10.3390/pharmaceutics17101340)
Supplement: Supplementary file 1 [file pharmaceutics-17-01340-s001.zip › pharmaceutics-3819623-supplementary.pdf]

PHARMACEUTICS

Supplementary Information associated with the paper

Silver(I)-Complexes Bearing S-Alkyl Thiosalicylic Acid Derivatives: DNA/BSA Binding and Antitumor Activity In Vitro and In Vivo

Jovana Marinković [1], Milena Jurišević [2,3*], Marina Jovanović [2,4], Miloš Milosavljević [5], Ivan Jovanović [2,6], Snežana Jovanović Stević [3], Marina Vesović [3*], Miloš Nikolić [3], Nikola Nedeljković [3], Ana Živanović [3], Dušan Tomović [3], Andriana Bukonjić [3], Gordana Radić [3] and Nevena Gajović [2]

[1] Faculty of Medicine of the Military Medical Academy, University of Defence, Crnotravska 17, 11000 Beograd, Serbia; jovana.eko@gmail.com (J.M.)
[2] Center for molecular medicine and stem cell research, Faculty of Medical Sciences, University of Kragujevac, Svetozara Markovića 69, 34000 Kragujevac, Serbia; gajovicnevena@yahoo.com (N.G.); milena.jurisevic13@gmail.com (M.J.); marina_jovanovic@rocketmail.com (M.J.); ivanjovanovic77@gmail.com (I.J.)
[3] Department of Pharmacy, Faculty of Medical Sciences, University of Kragujevac, Svetozara Markovića 69, 34000 Kragujevac, Serbia; milena.jurisevic13@gmail.com (M.J.); snezanaj@kg.ac.rs (S.J.); marina.vesovic@fmn.kg.ac.rs (M.V.); milos.nikolic@fmn.kg.ac.rs (M.N.); nikola.nedeljkovic@fmn.kg.ac.rs (N.N); ana.zivanovic@fmn.kg.ac.rs (A.Ž.); andriana.bukonjic@hotmail.com (A.B.); dusantomovic88@hotmail.com (D.T.); vasic_gordana@yahoo.com (G.R.)
[4] Department of Otorhinolaryngology, Faculty of Medical Sciences, University of Kragujevac, Serbia; marina_jovanovic@rocketmail.com (M.J.)
[5] Department of Pathology, University Medical Center Kragujevac, Kragujevac, Serbia; m.milosavljevic77@gmail.com (M.M.)
[6] Faculty of medicine, University of East Sarajevo, Studentska 5, 73300 Foca, Bosnia and Herzegovina; ivanjovanovic77@gmail.com (I.J.)
* Correspondence: M.V. marina.vesovic@fmn.kg.ac.rs and M.J. milena.jurisevic13@gmail.com

Contents

NMR and FTIR spectra of free ligands and complexes

Figure S1. Absorption spectra of **C2-C5** at 25 °C in PBS buffer upon addition of CT-DNA. [complex] = 8×10^{-6} M, [DNA] = $(0\text{-}6.4) \times 10^{-6}$ M. Arrow shows the change of the absorbance with the increase of DNA concentration. Inset: plot of [DNA]/($\varepsilon_A - \varepsilon_f$) *vs.* [DNA].

Figure S2. Relative viscosity $(\eta/\eta_0)^{1/3}$ of CT-DNA (8 μM) in PBS buffer solution with the addition of increasing amounts (r) of **C2** and **C4** complexes.

Figure S3. Emission spectra of EB bound to DNA in the presence of **C1, C2, C3** and **C5**. [EB] = 8.52×10^{-5} M; [DNA] = 8.52×10^{-5} M; [complex] = $(0\text{-}6.82) \times 10^{-5}$; λ_{ex} = 527 nm. Arrows show the intensity changes upon increasing the concentration of complex. Inset graph: Plot of I_0/I *vs*. [Q].

Figure S4. Emission spectra of BSA in the presence of **C2-C5**. [BSA] = 2 μM, [complex] = 0-8 μM, λ_{ex} = 295 nm. Arrows show the intensity changes upon increasing the concentrations of complex. Inset graph: Plot of I_0/I *vs.* [Q].

Figure S5. Scatchard plots for **C1-C5** complexes.

Figure S6. Dose-dependent cytotoxicity of silver(I)-complexes with S-alkyl derivatives of thiosalicylic acid. Effect of **C1-C5** on viability of 4T1 (A), MDA-MB-468(B), CT26(C), HCT116(D), LLC1(E), A549(F), mMSC(G) and MRC-5(H) cells after period of incubation of 24h and 48h analyzed with the MTT assay. All data are presented as mean values ± SD from three independent experiments performed in triplicates.

Figure S7. Dose-dependent cytotoxicity S-alkyl derivatives of thiosalicylic acid. Effect of **L1-L5** on viability of 4T1 (A), MDA-MB-468(B), CT26(C), HCT116(D), LLC1(E), A549(F), mMSC(G) and MRC-5(H) cells after period of incubation of 24h and 48h analyzed with the MTT assay. All data are presented as mean values ± SD from three independent experiments performed in triplicates.

Figure S8. CT26 and HCT116 cells underwent apoptosis after **C2** treatment. Apoptotic rates of **C2** treated (0.5 μM for 24 hours) as well as untreated CT26 and HCT116 cells, were determined by flow cytometry using Annexin V (FITC) and PI double staining. The data are shown as averages ± SD of a 3 independent experiment. Mann-Whitney U test* $p<0.05$ compared with the untreated group.

Figure S9. Relationship between DNA-binding constants (Kb) and IC_{50} values.

In the spectra of S-alkyl derivatives of thiosalicylic acid (free ligands), a broad and very intense band in the region of 2500 to 3000 cm^{-1}, attributed to O-H valence vibration, is observed. Additionally, an intense band at about 740 cm^{-1} confirms the presence of 1,2-disubstituted benzene indicating ortho substitution on the aromatic ring. The C=O valence vibration gives a strong absorbance band observed in the region of 1674 to 1682 cm^{-1} (1674 cm^{-1} for **L1**, 1682 cm^{-1} for **L2**, 1678 cm^{-1} for **L3**, 1674 cm^{-1} for **L4**, and 1674 cm^{-1} for **L5**), while the C-O valence vibration couples with the O-H deformation vibration , appearing asa weak band at 1412 cm^{-1} for **L1**, 1414 cm^{-1} for **L2**, 1405 cm^{-1} for **L3**, 1408 cm^{-1} for **L4**, and 1412 cm^{-1} for **L5** along with a slightly more intense band at 1255 cm^{-1} for **L1**, 1252 cm^{-1} for **L2**, 1257 cm^{-1} for **L3**, 1250 cm^{-1} for **L4**, and 1255 cm^{-1} for **L5**. The presence of these bands clearly confirms the existence of the carboxyl group.

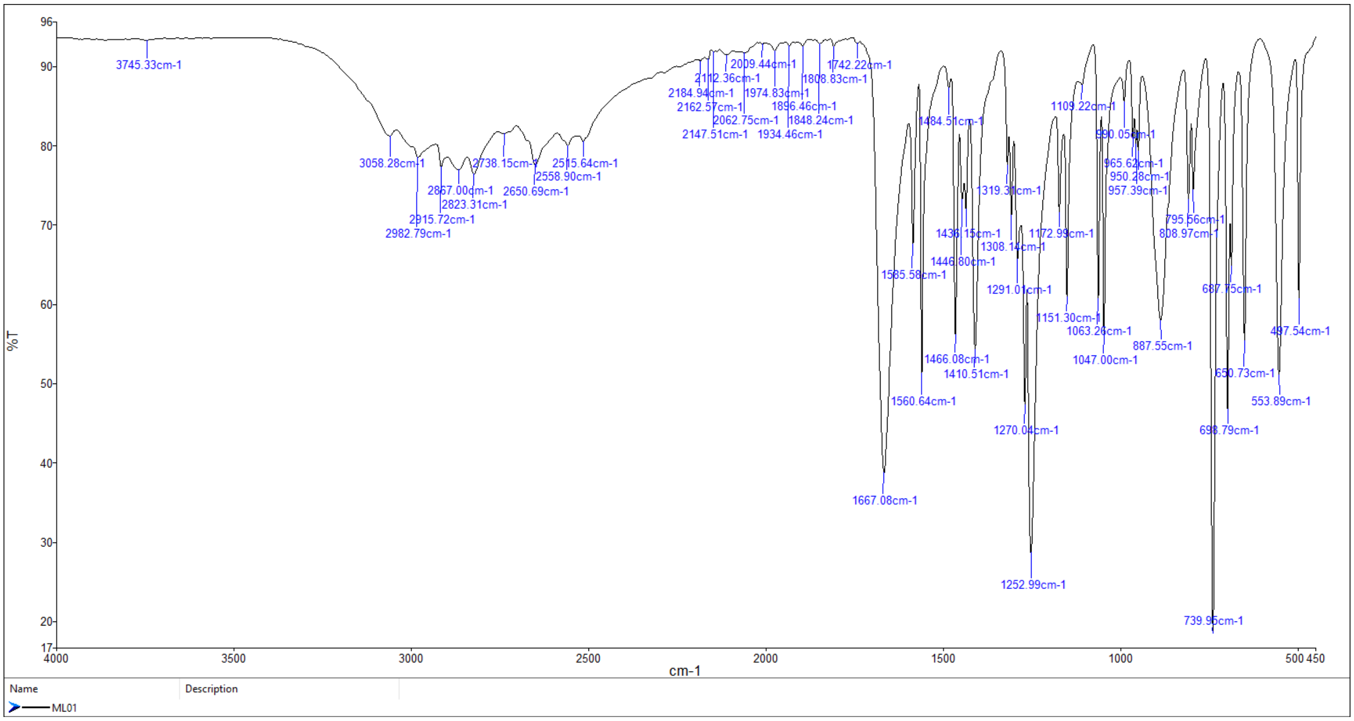

IR spectrum of the S-methyl derivative of thiosalicylic acid

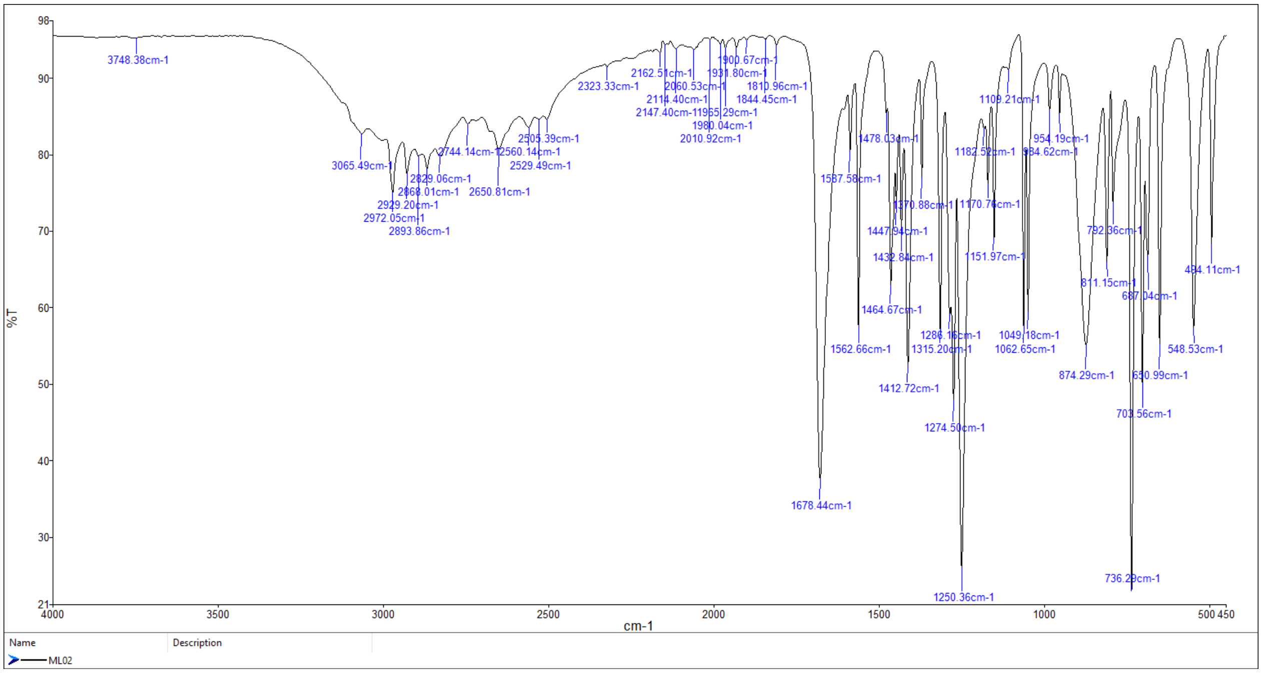

IR spectrum of the S-ethyl derivative of thiosalicylic acid

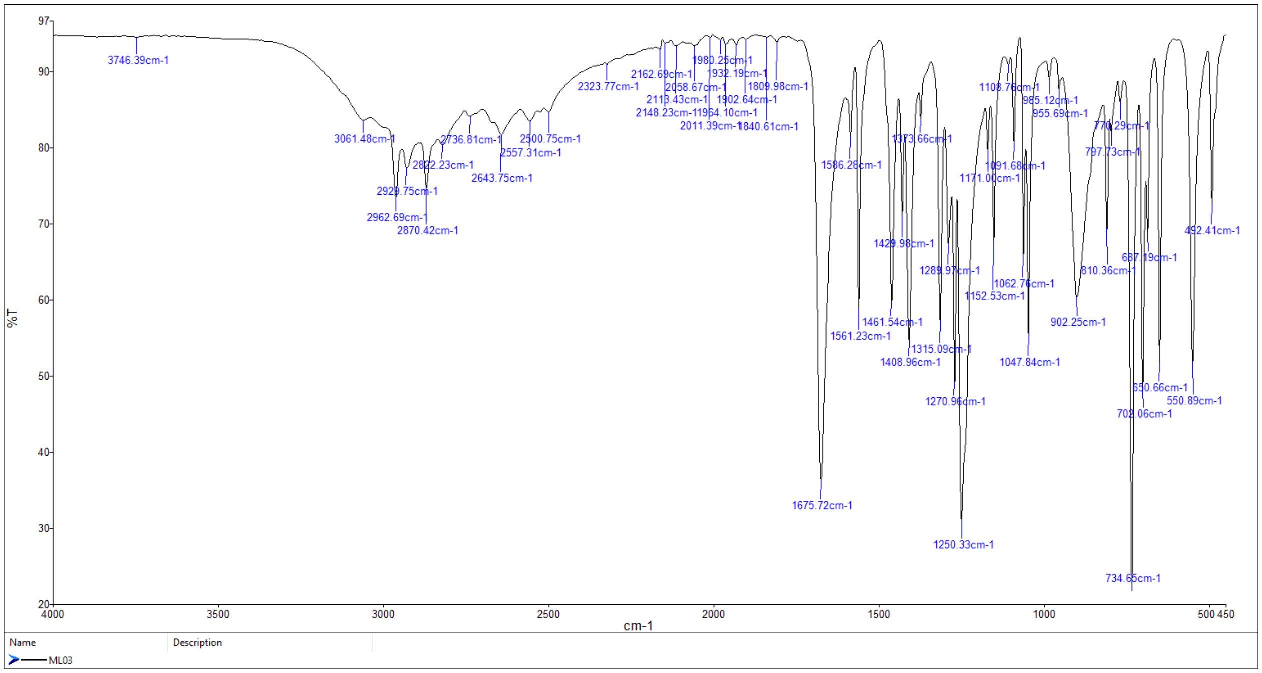

IR spectrum of the S-propyl derivative of thiosalicylic acid

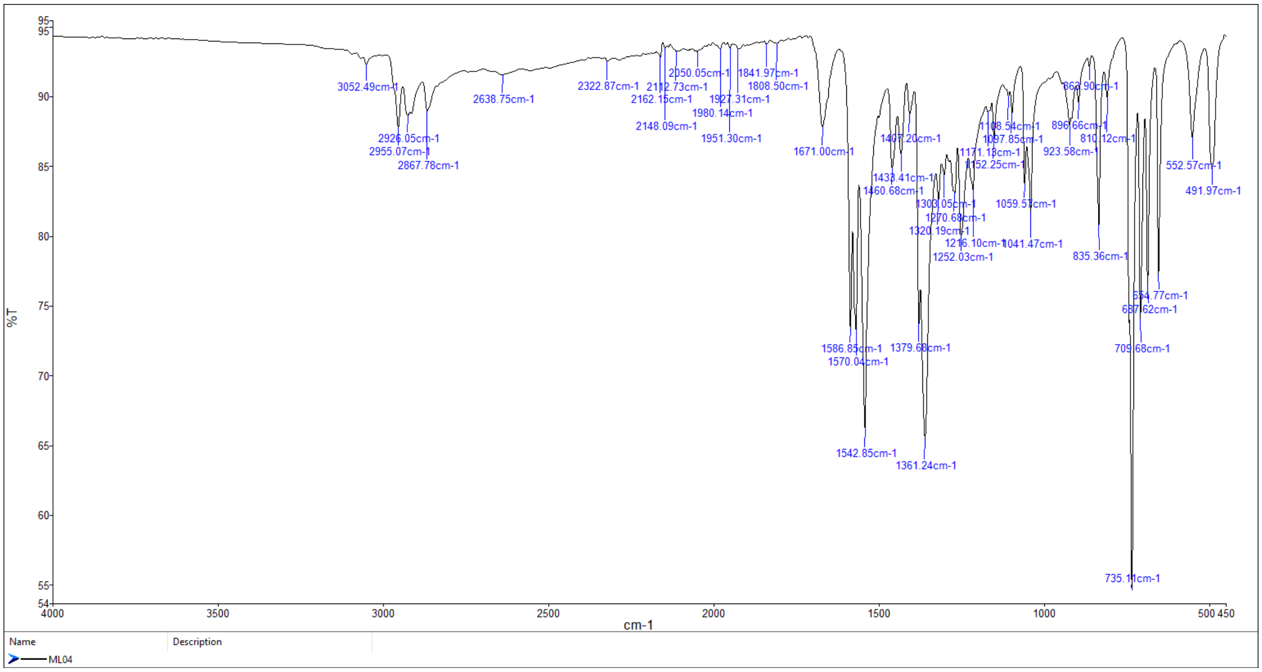

IR spectrum of the S-butyl derivative of thiosalicylic acid

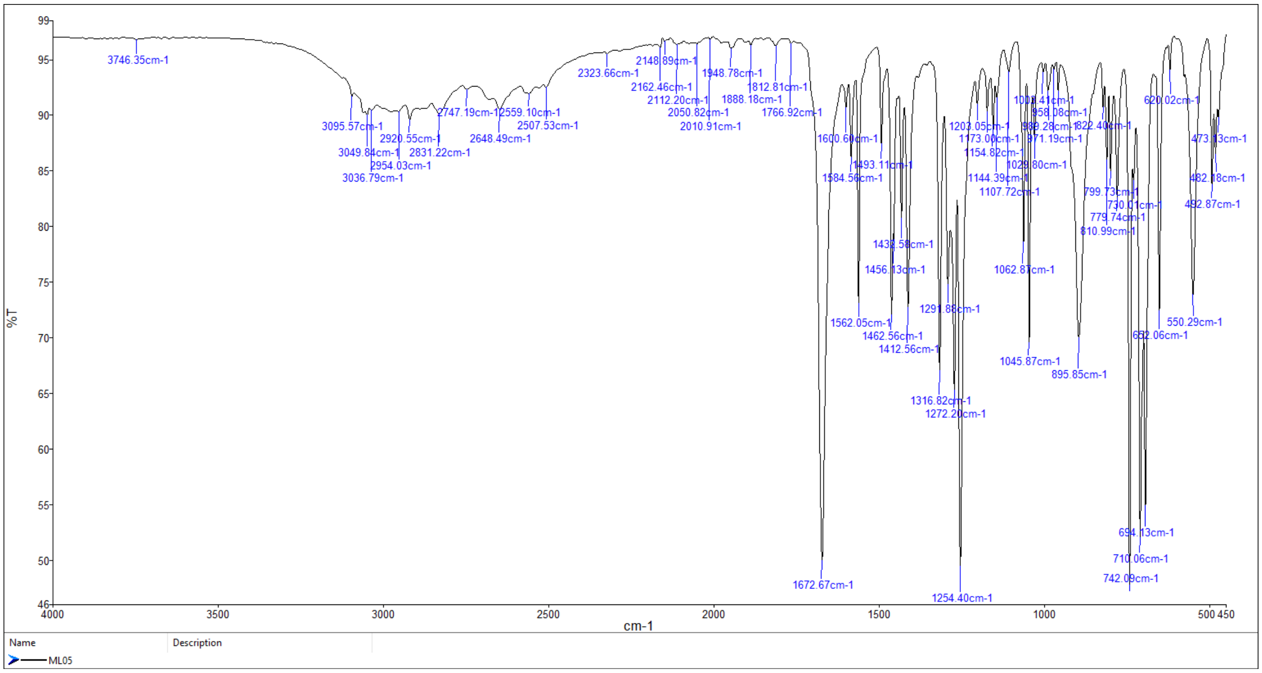

IR spectrum of the S-benzyl derivative of thiosalicylic acid

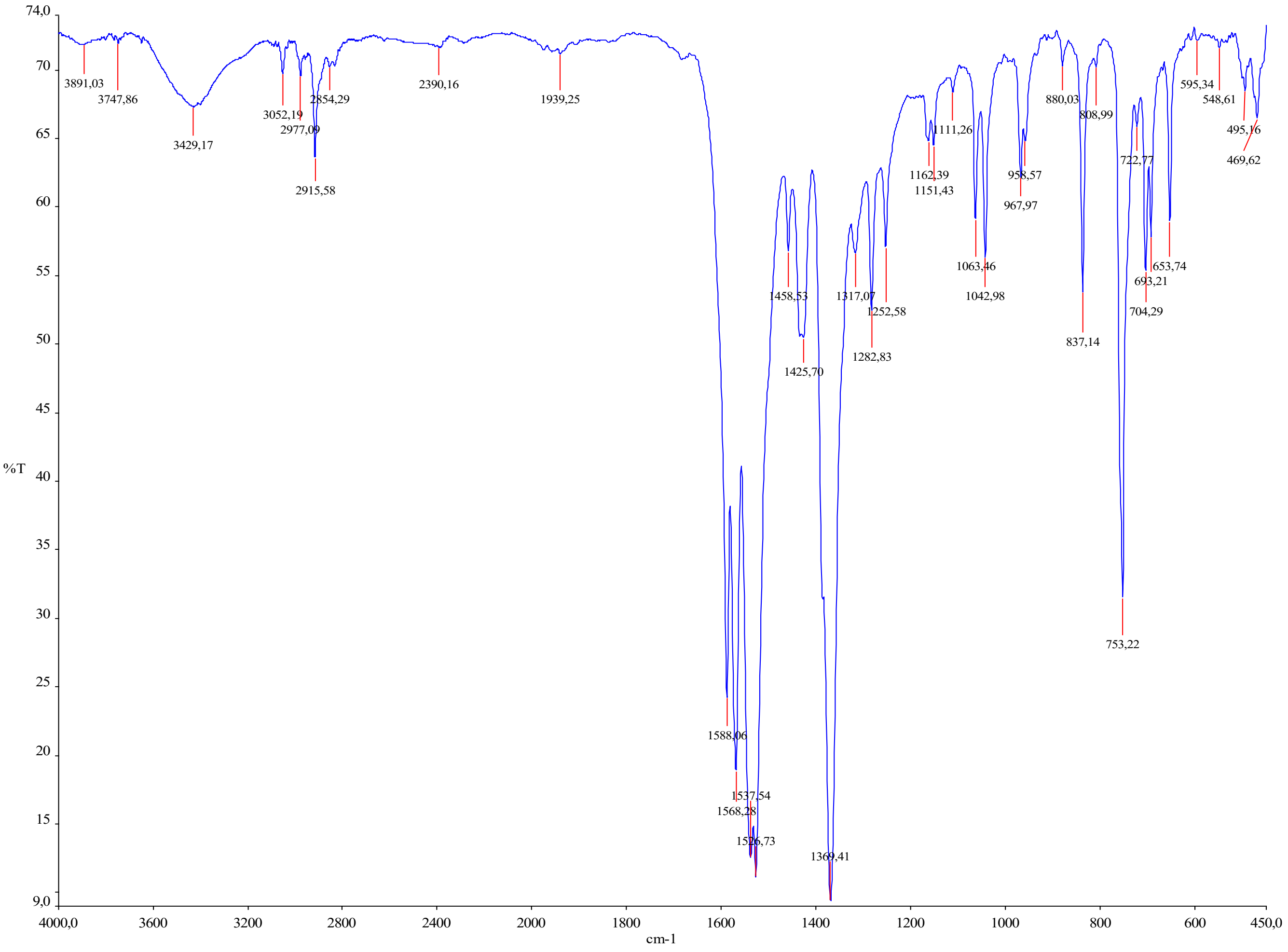

IR spectrum of the complex [Ag_2(S-methyl-thiosal)$_2$]

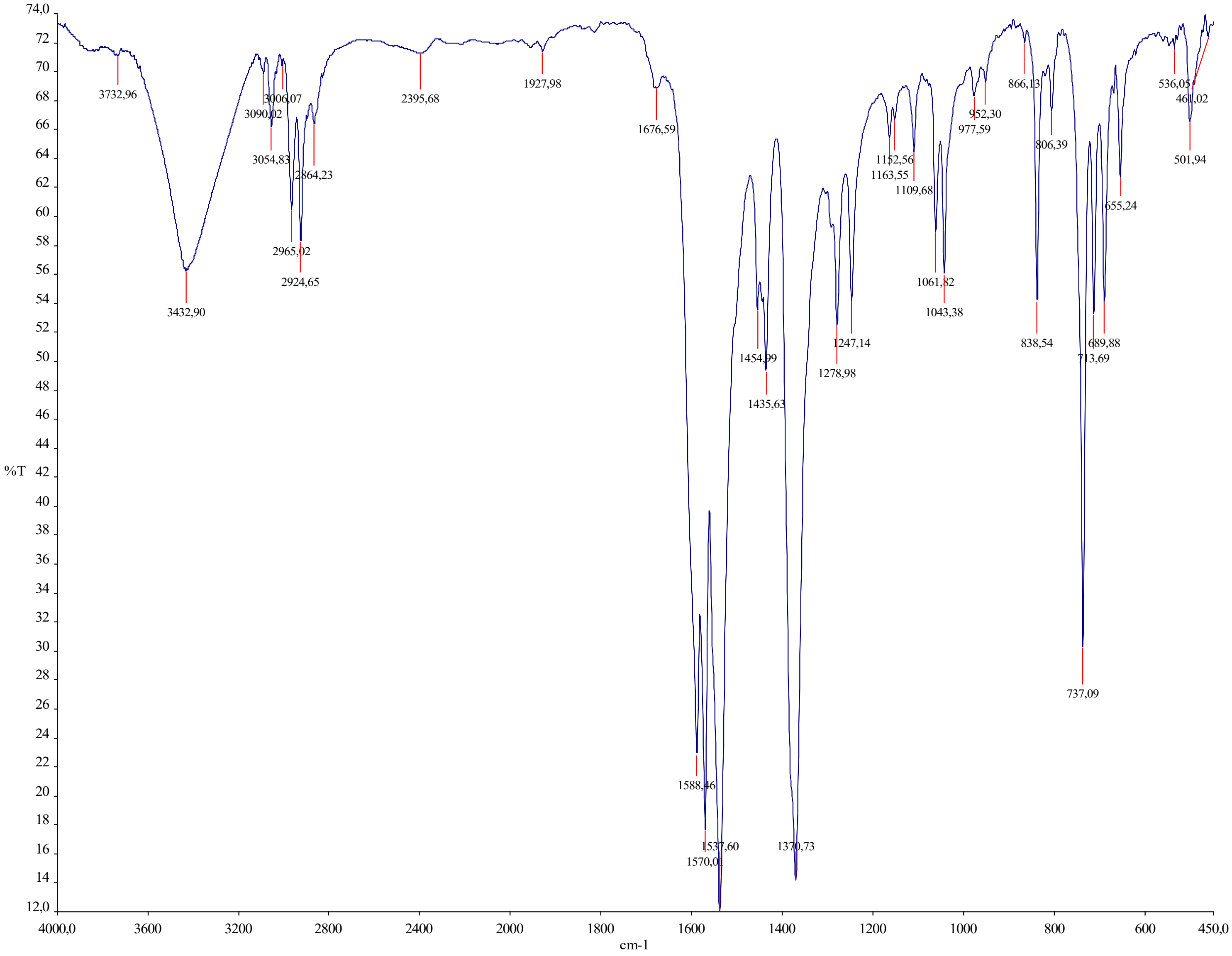

IR spectrum of the complex [Ag_2(S-ethyl-thiosal)$_2$]

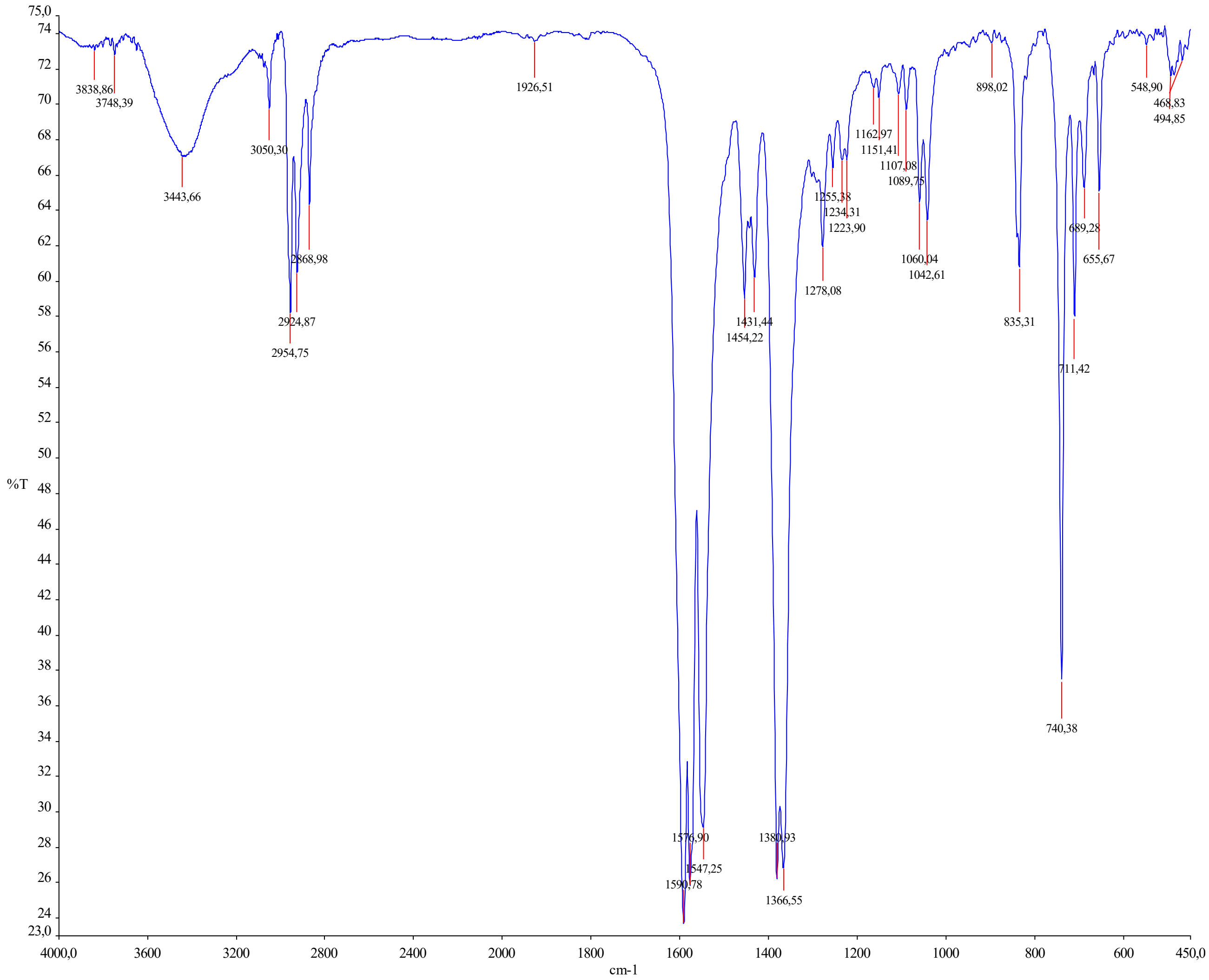

IR spectrum of the complex [Ag_2(S-propyl-thiosal)$_2$]

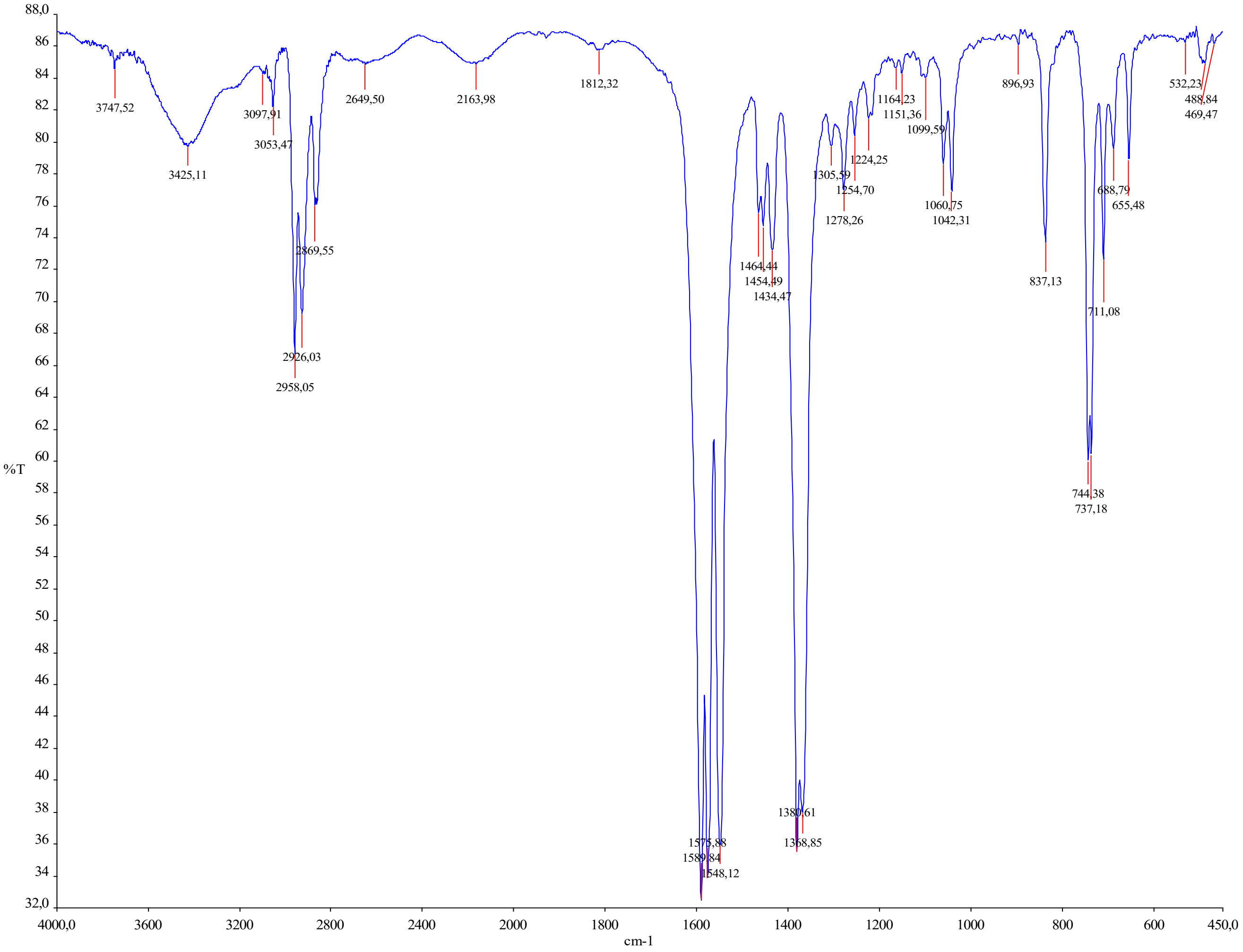

IR spectrum of the complex [Ag_2(S-butyl-thiosal)$_2$]

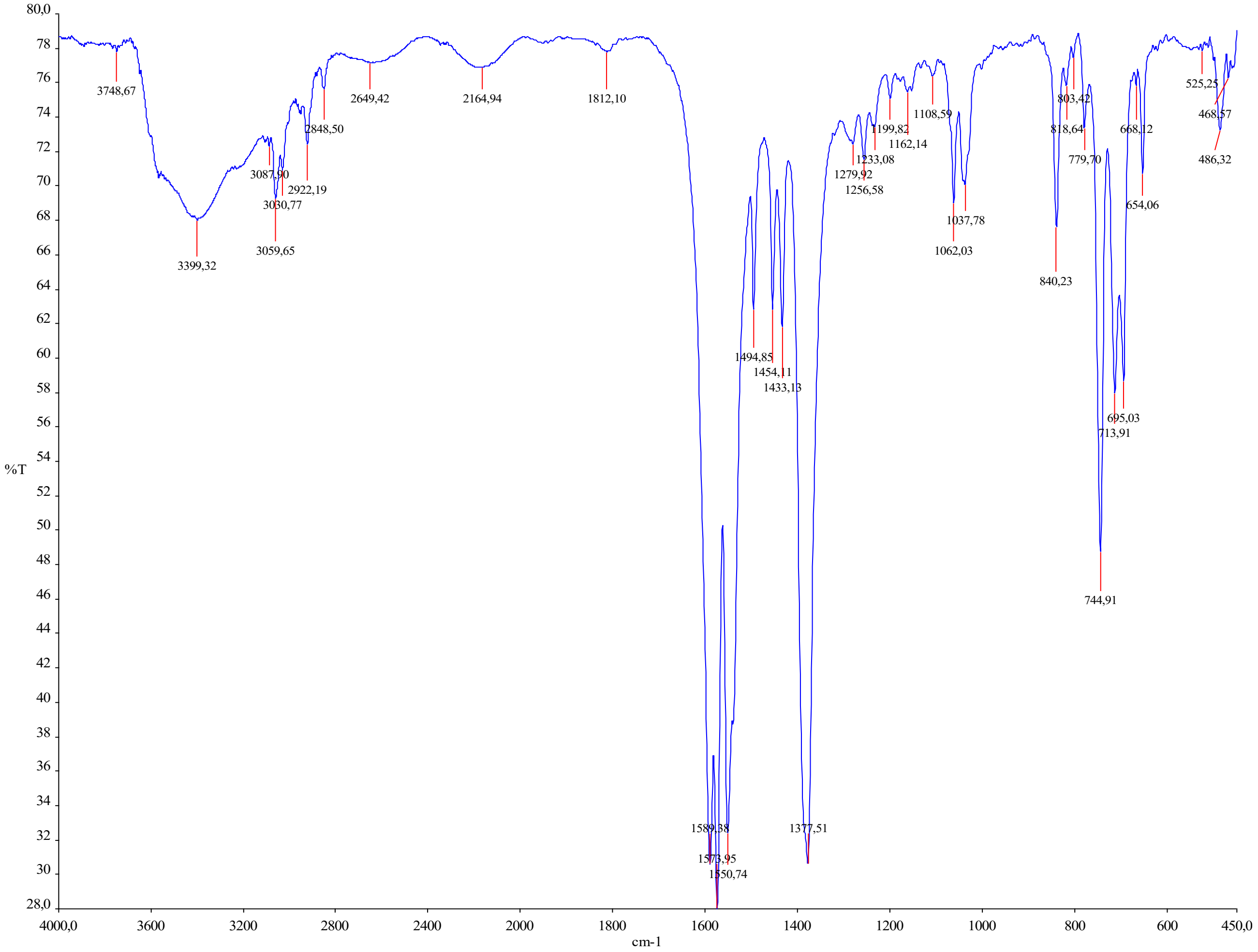

IR spectrum of the complex [Ag_2(S-benzyl-thiosal)$_2$]

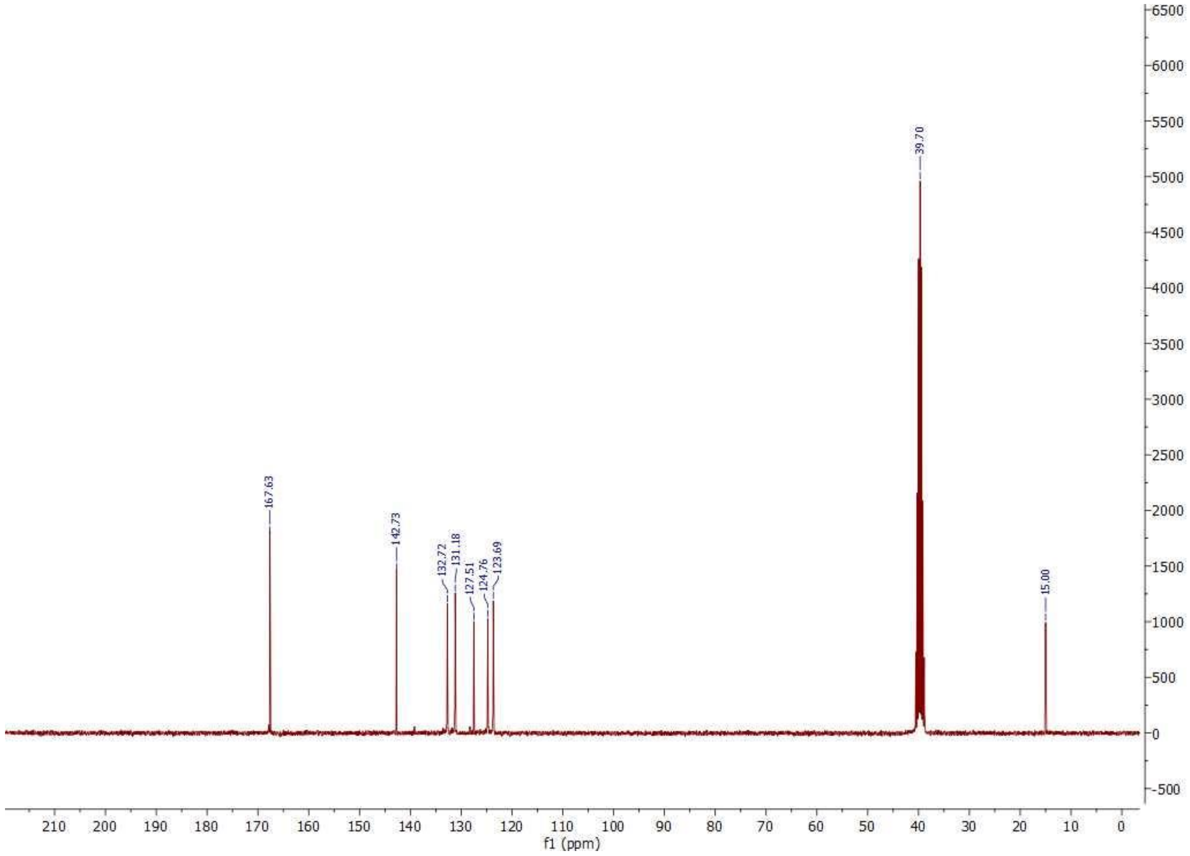

^{13}C NMR spectrum of the S-methyl derivative of thiosalicylic acid

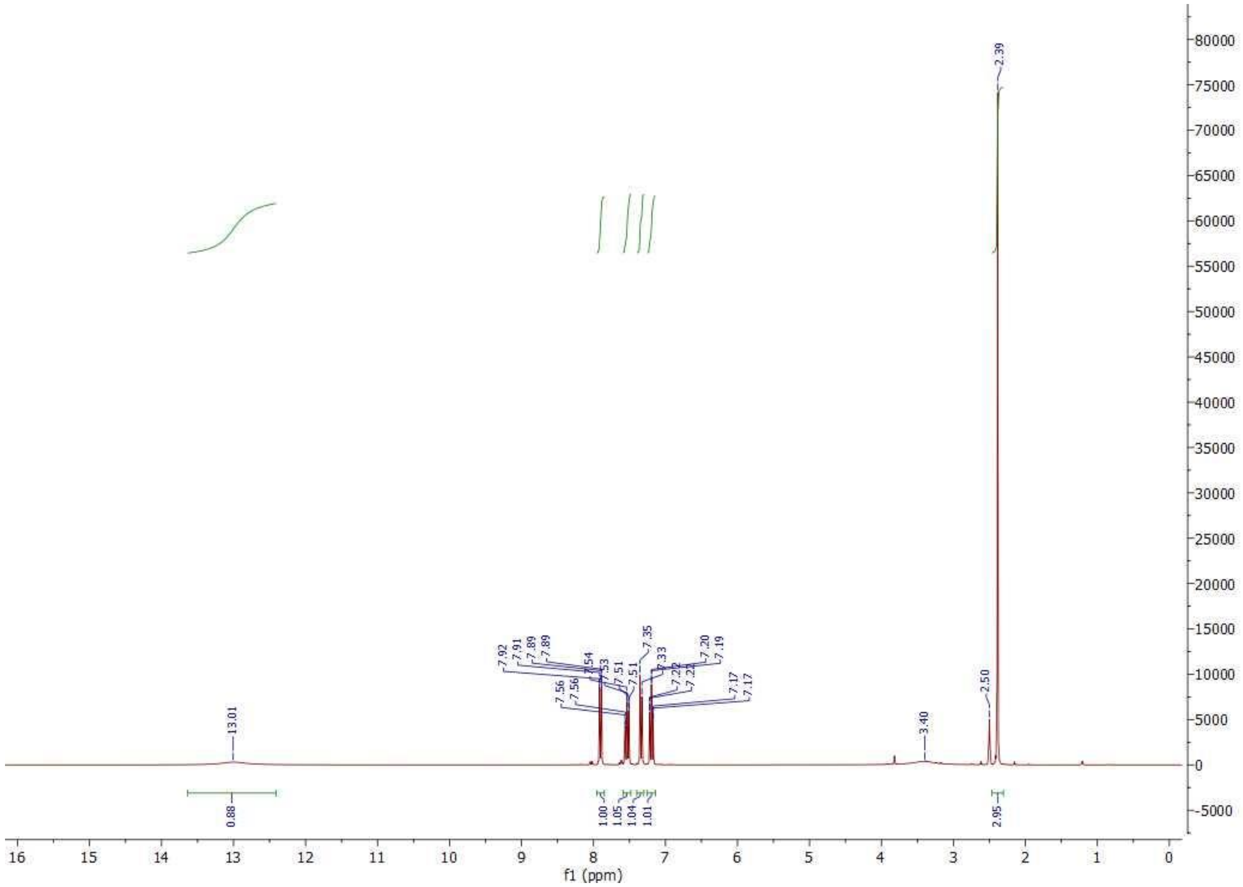

^{1}H NMR spectrum of the S-methyl derivative of thiosalicylic acid

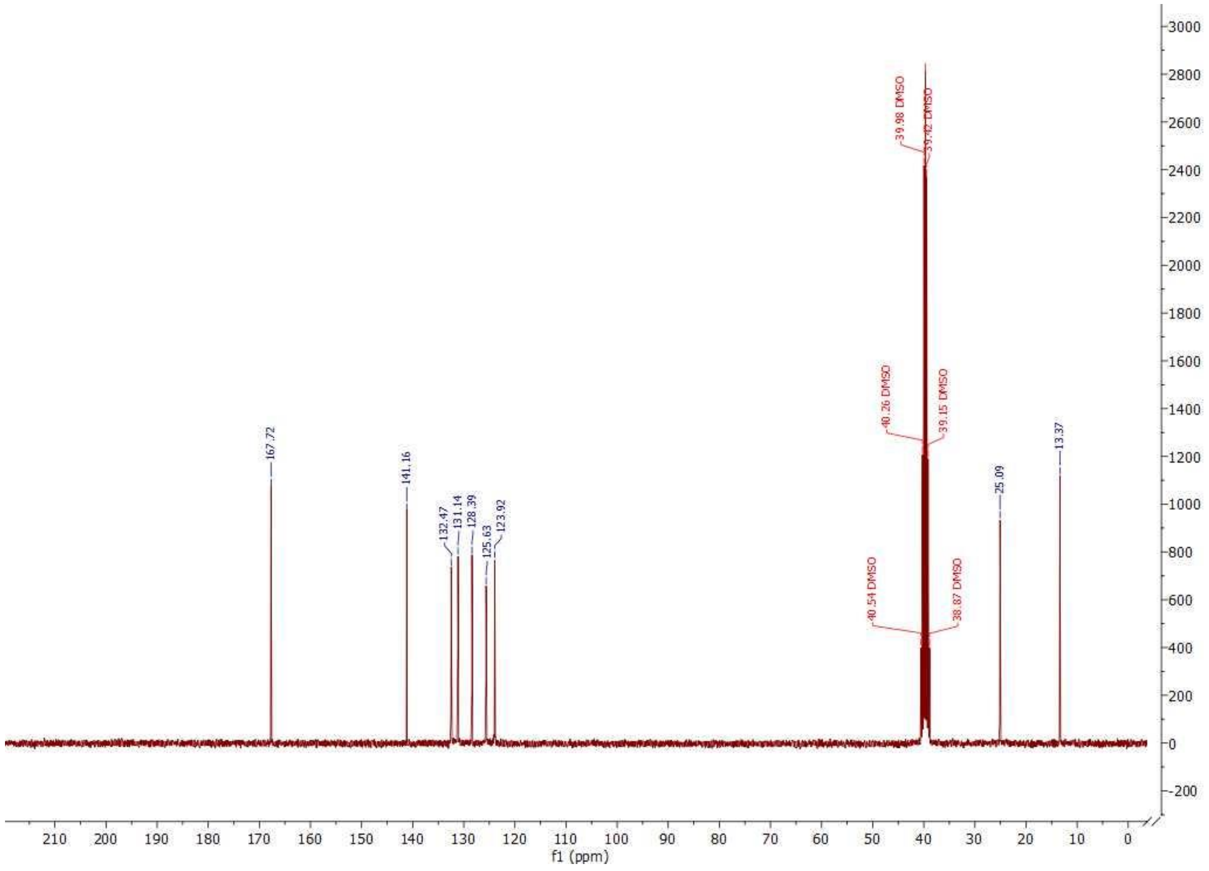

^{13}C NMR spectrum of the S-ethyl derivative of thiosalicylic acid

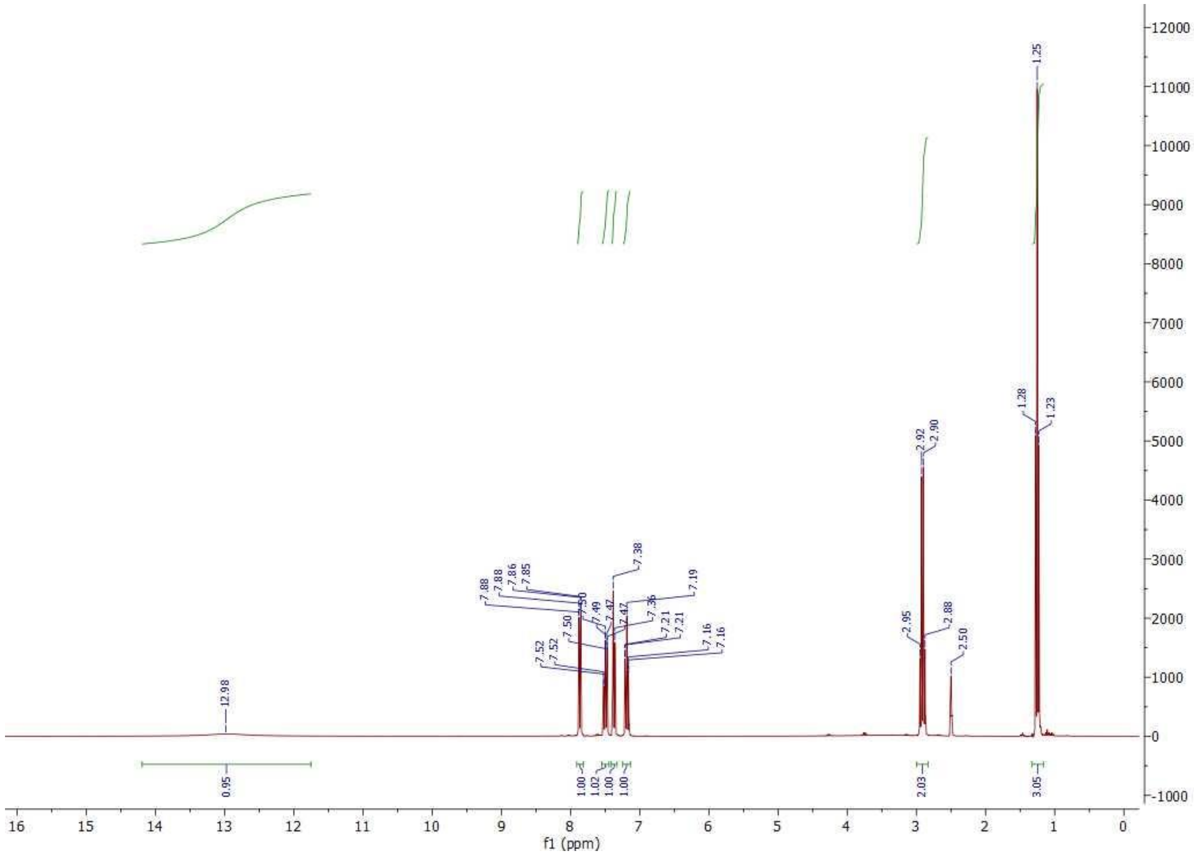

^{1}H NMR spectrum of the S-ethyl derivative of thiosalicylic acid

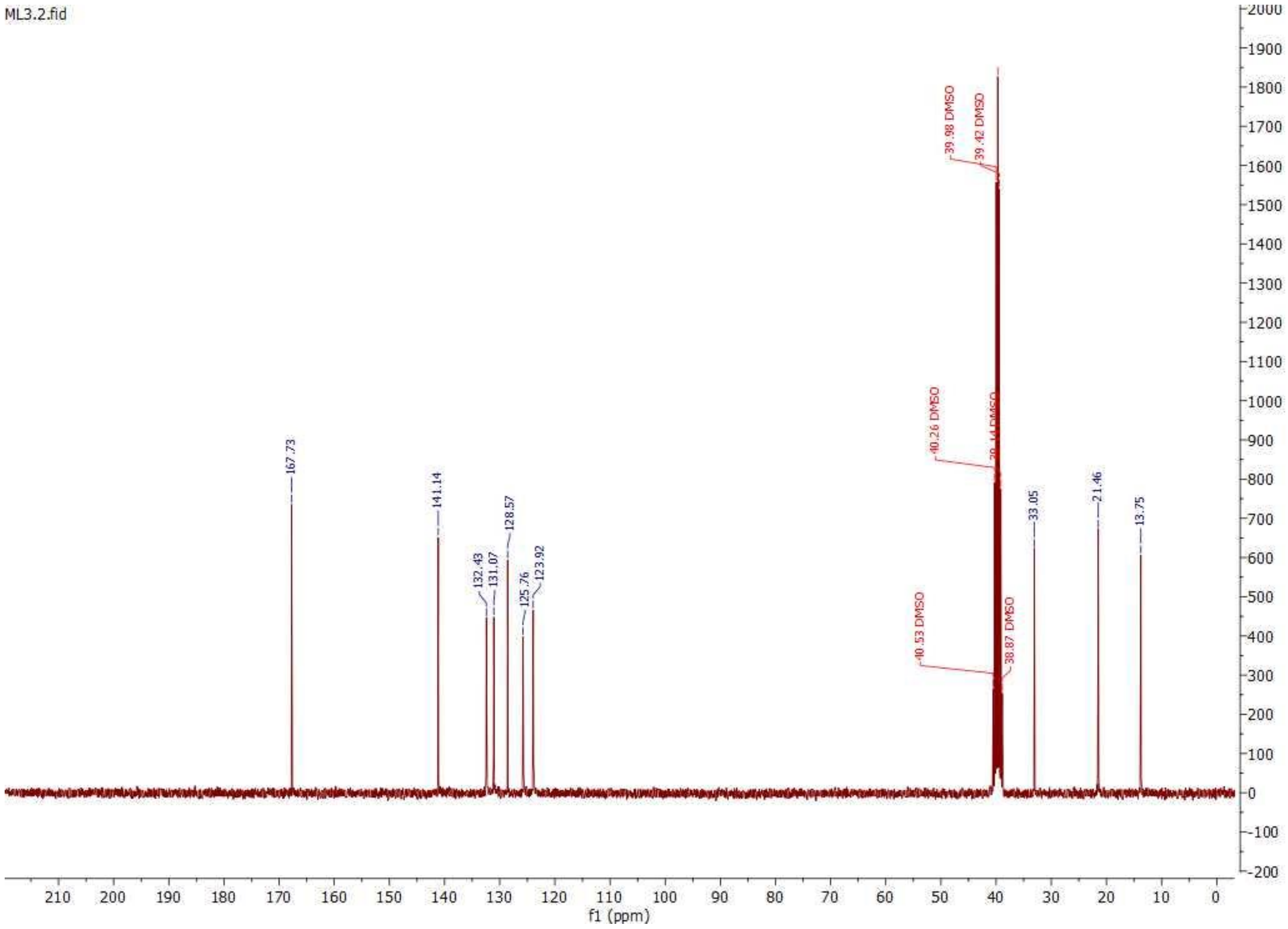

^{13}C NMR spectrum of the S-propyl derivative of thiosalicylic acid

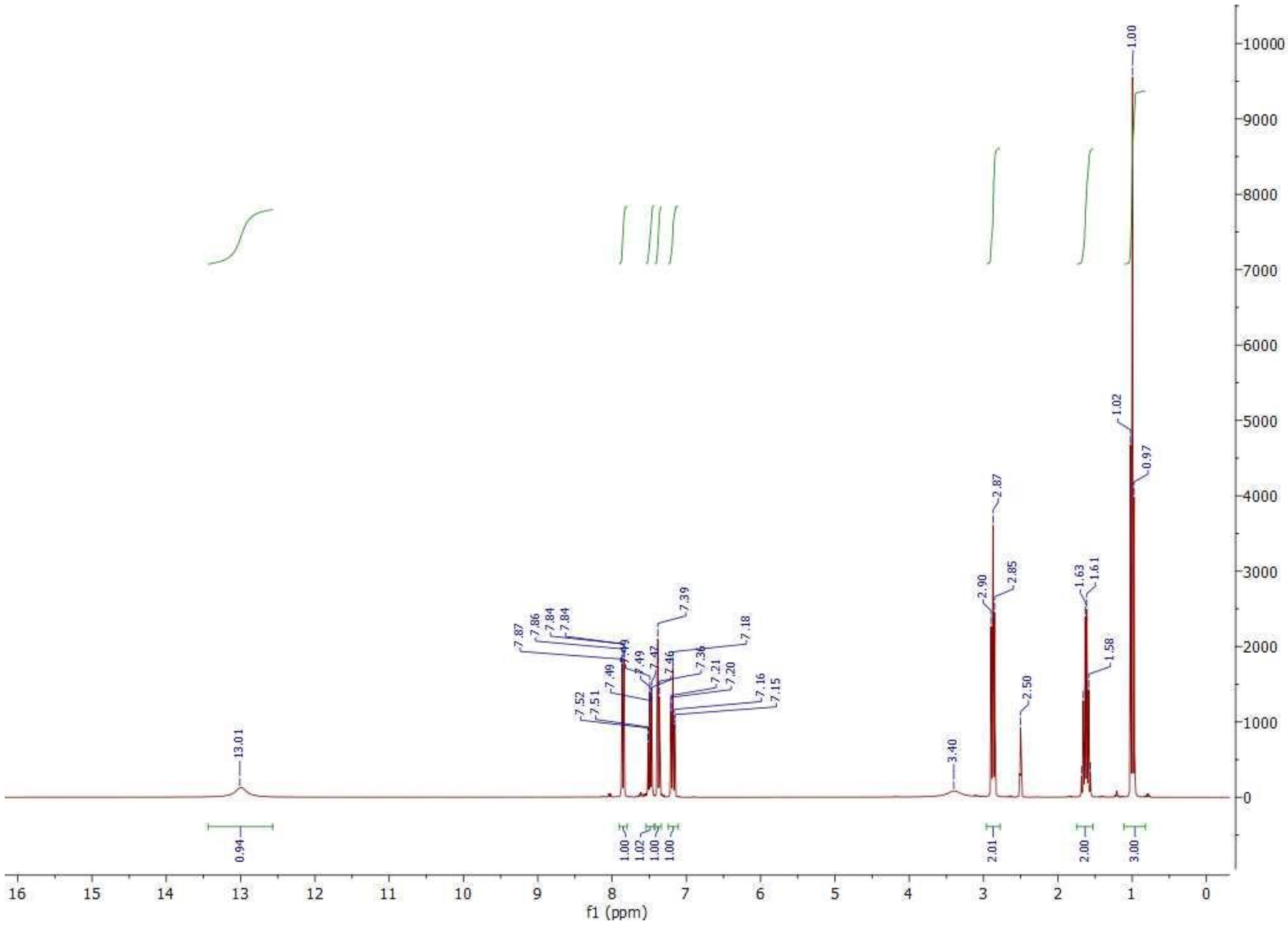

^{1}H NMR spectrum of the S-propyl derivative of thiosalicylic acid

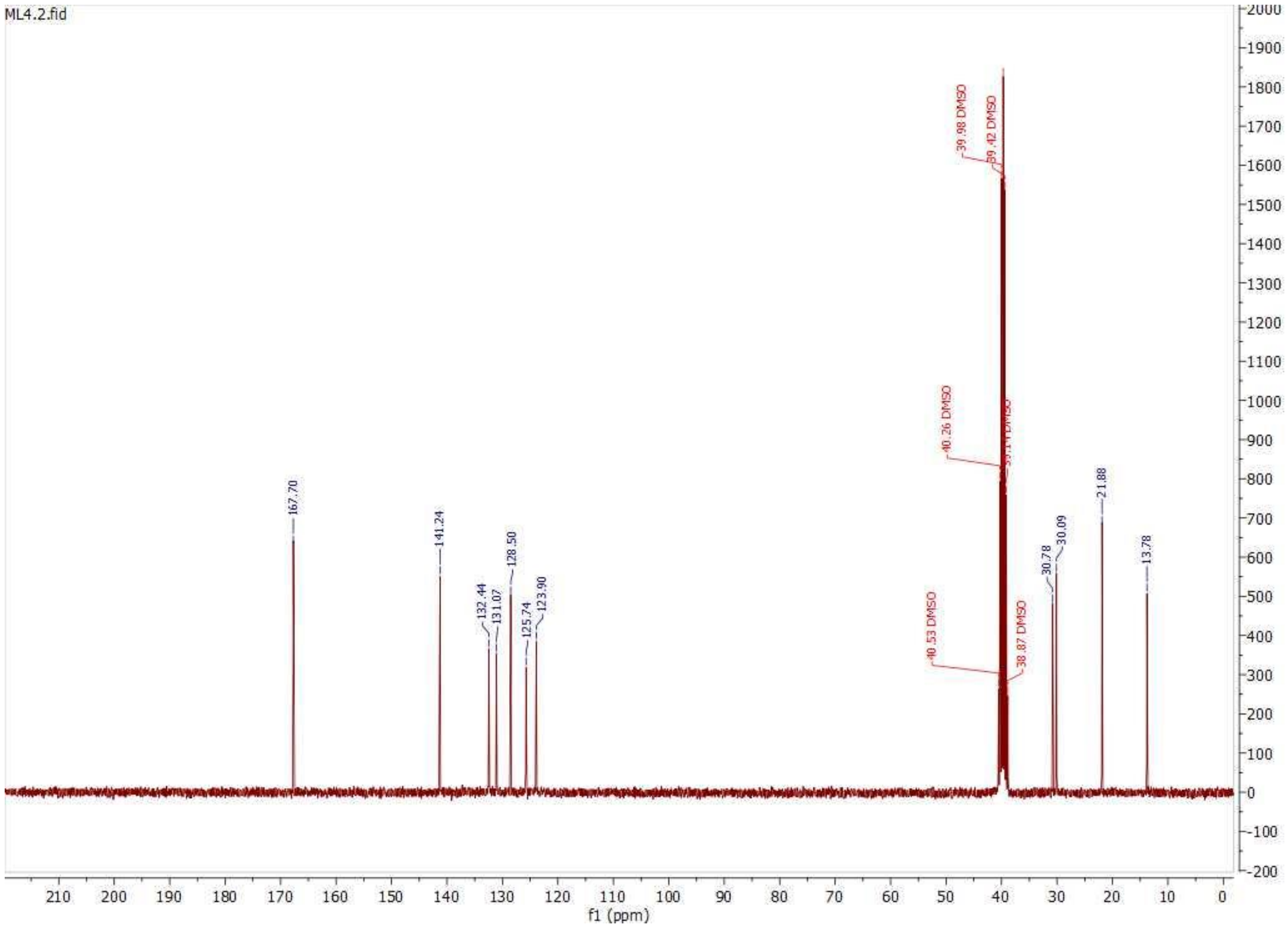

^{13}C NMR spectrum of the S-butyl derivative of thiosalicylic acid

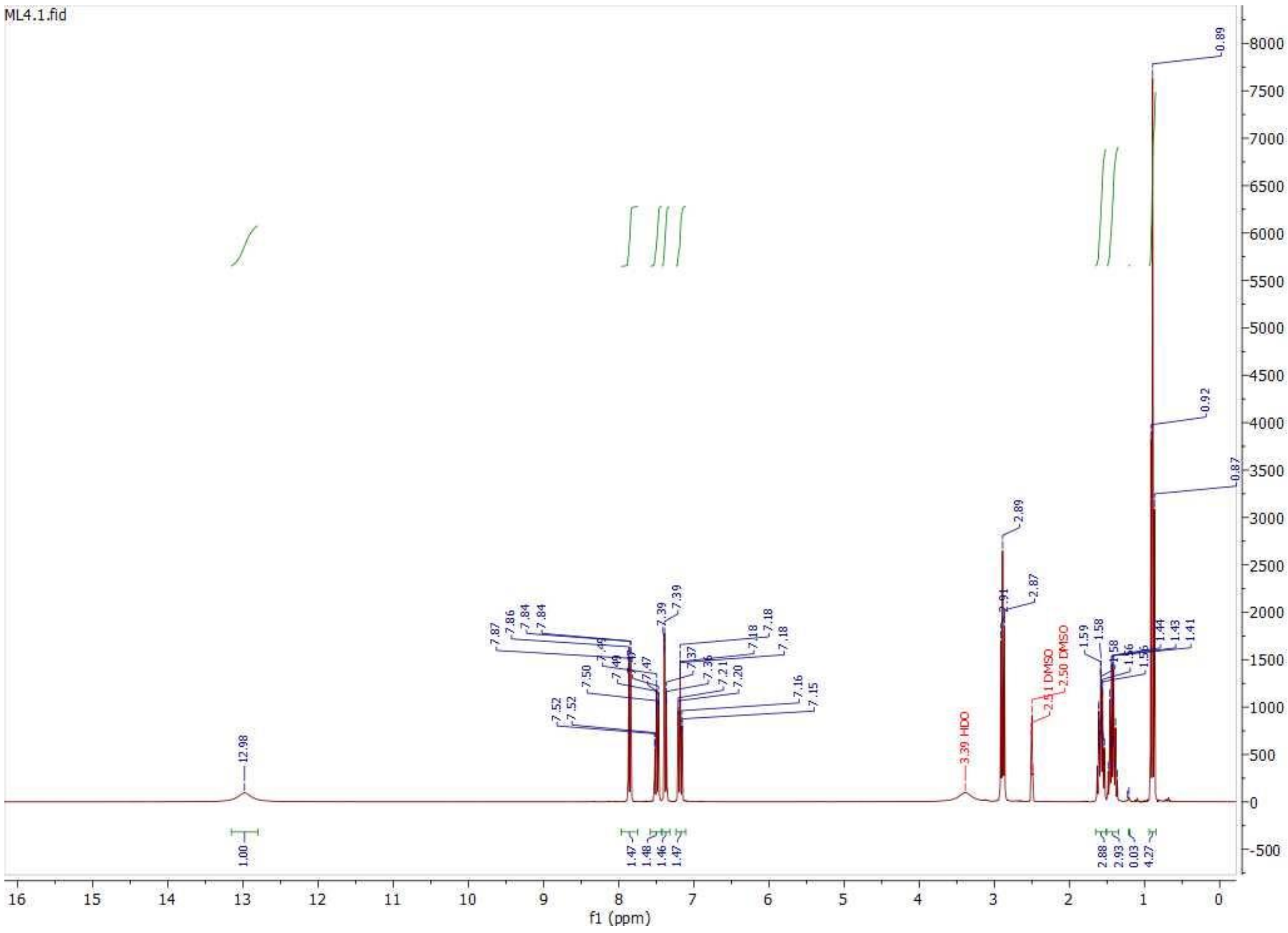

^{1}H NMR spectrum of the S-butyl derivative of thiosalicylic acid

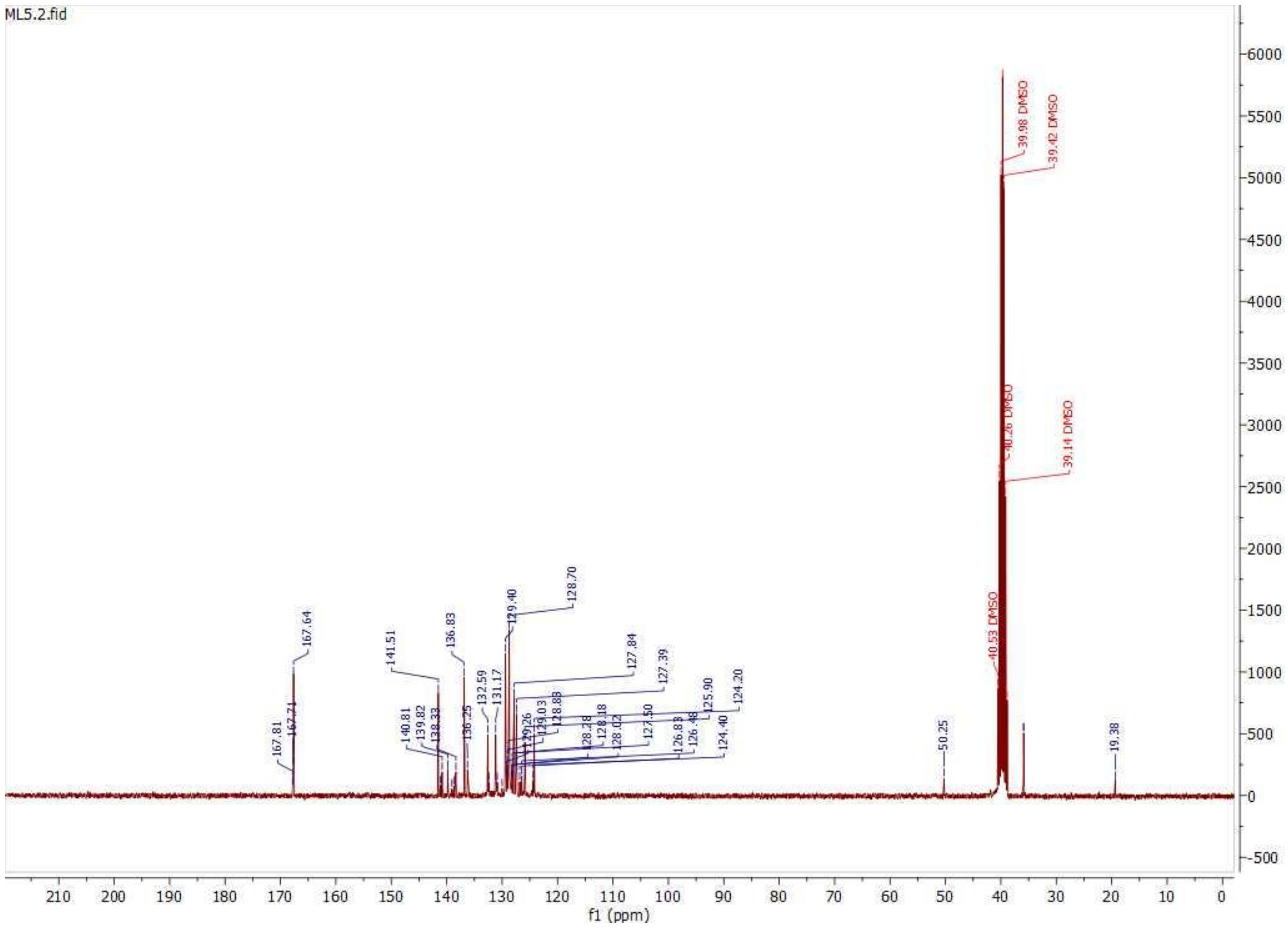

^{13}C NMR spectrum of the S-benzyl derivative of thiosalicylic acid

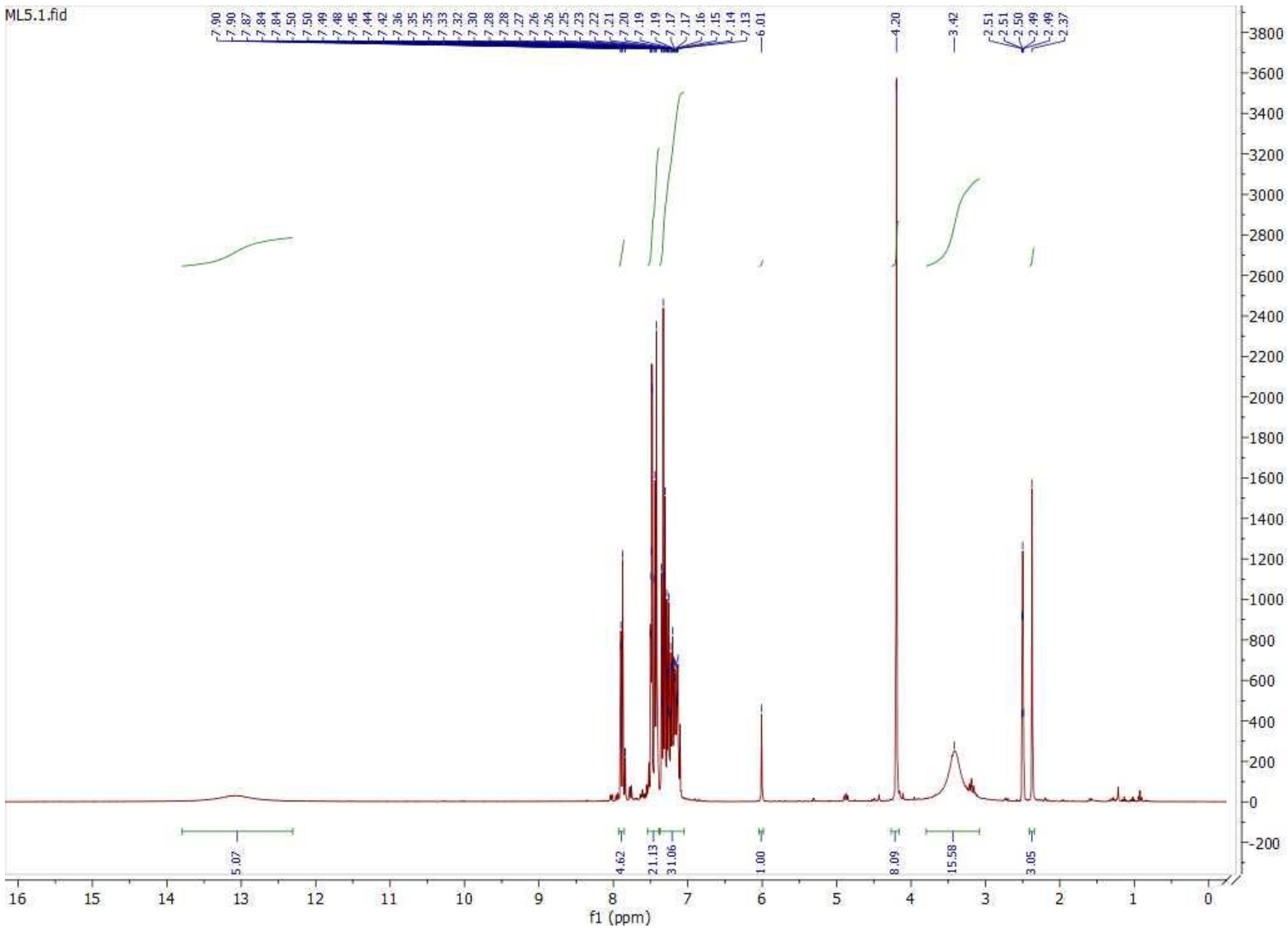

^{1}H NMR spectrum of the S-benzyl derivative of thiosalicylic acid

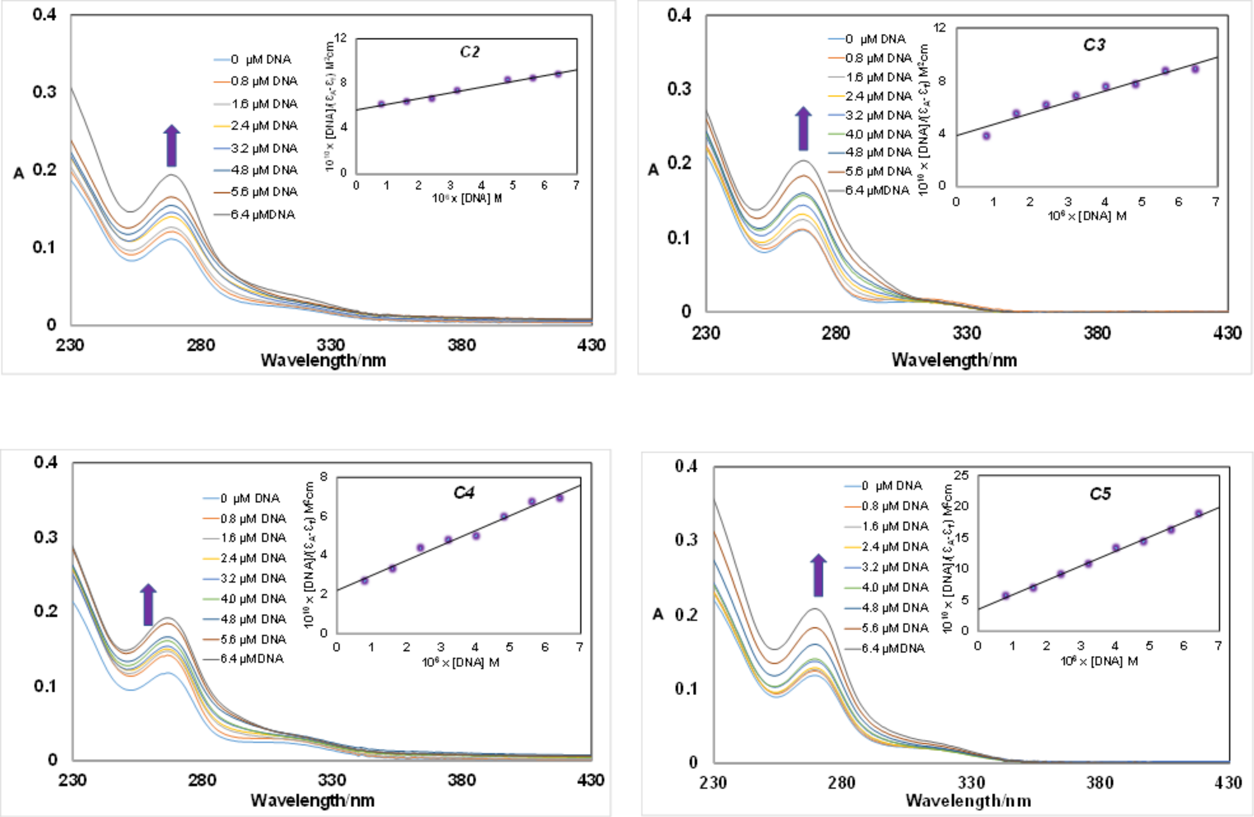

Figure S1. Absorption spectra of **C2-C5** at 25 °C in PBS buffer upon addition of CT-DNA. [complex] = 8 × 10^{-6} M, [DNA] = (0-6.4) × 10^{-6} M. Arrow shows the change of the absorbance with the increase of DNA concentration. Inset: plot of [DNA]/($\varepsilon_A - \varepsilon_f$) *vs.* [DNA].

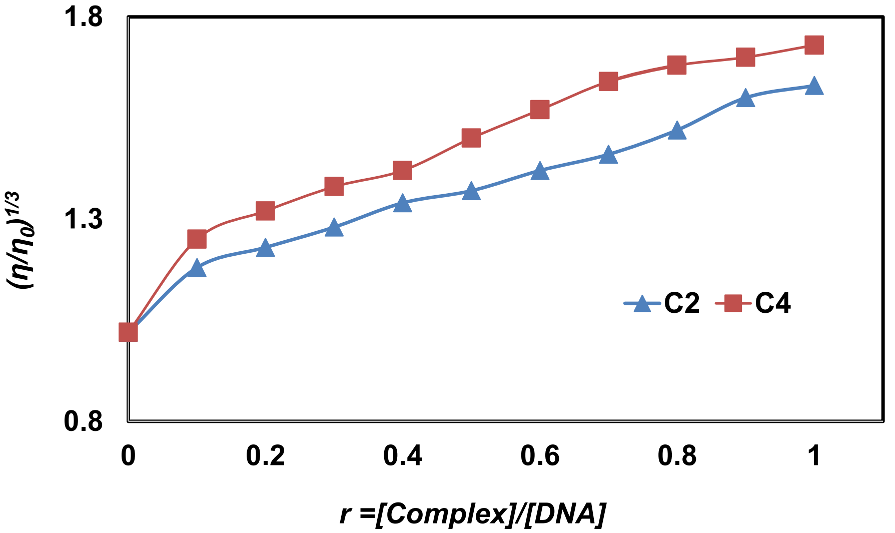

Figure S2. Relative viscosity $(\eta/\eta_0)^{1/3}$ of CT-DNA (8 μM) in PBS buffer solution with the addition of increasing amounts (r) of C2 and C4 complexes.

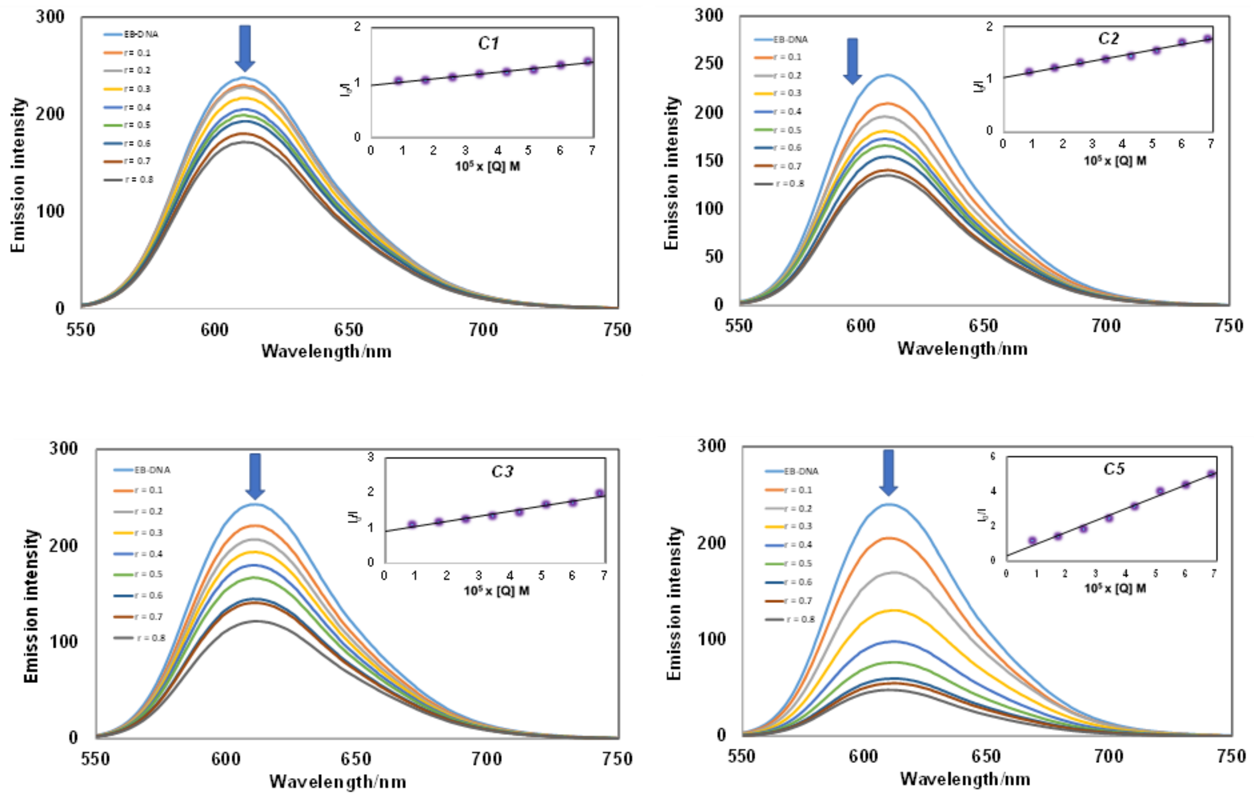

Figure S3. Emission spectra of EB bound to DNA in the presence of **C1, C2, C3** and **C5**. [EB] = 8.52×10^{-5} M; [DNA] = 8.52×10^{-5} M; [complex] = (0-6.82) × 10^{-5}; λ_{ex} = 527 nm. Arrows show the intensity changes upon increasing the concentration of complex. Inset graph: Plot of I_0/I *vs*. [Q].

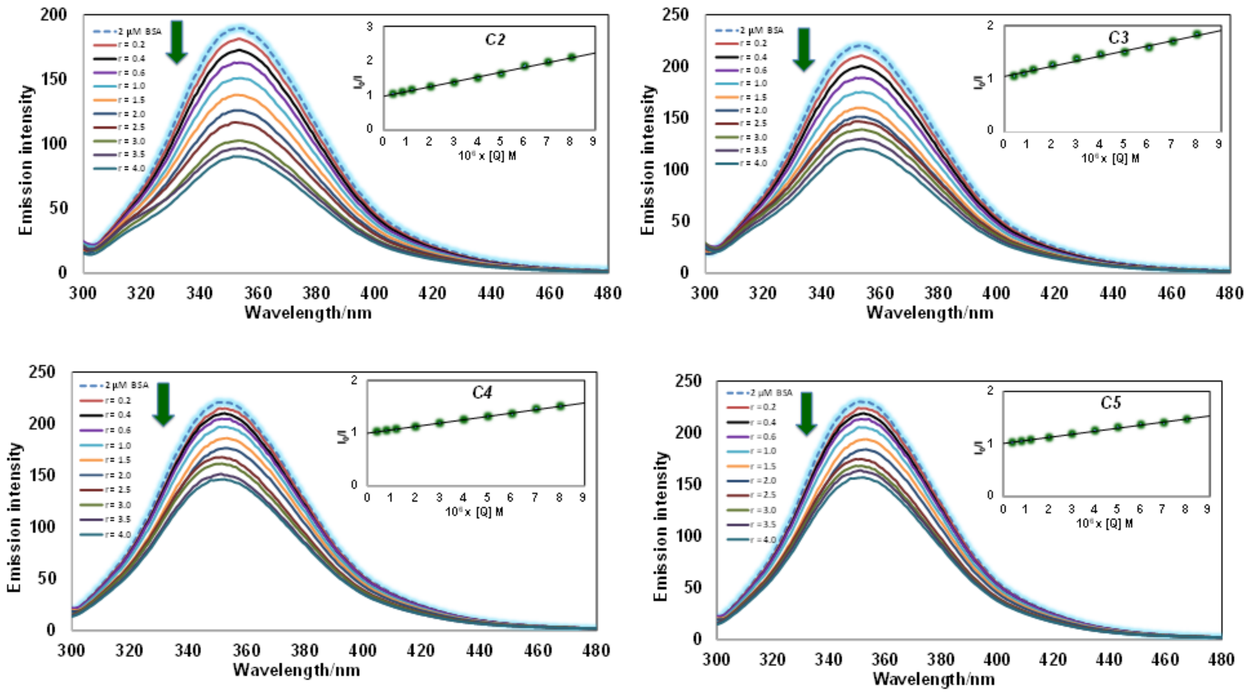

Figure S4. Emission spectra of BSA in the presence of **C2-C5**. [BSA] = 2 μM, [complex] = 0-8 μM, λ_{ex} = 295 nm. Arrows show the intensity changes upon increasing the concentrations of complex. Inset graph: Plot of I_0/I *vs.* [Q].

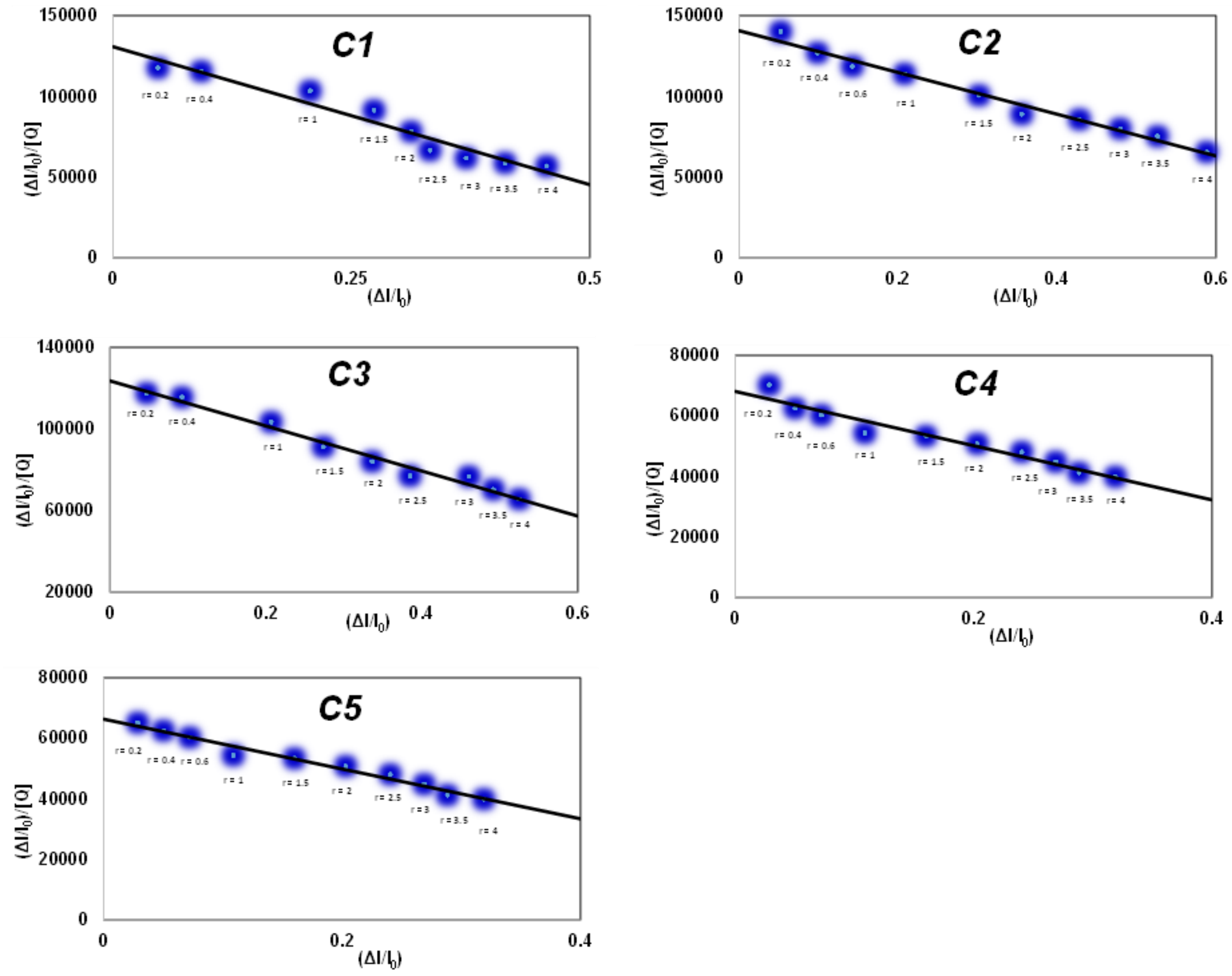

Figure S5. Scatchard plots for **C1-C5** complexes.

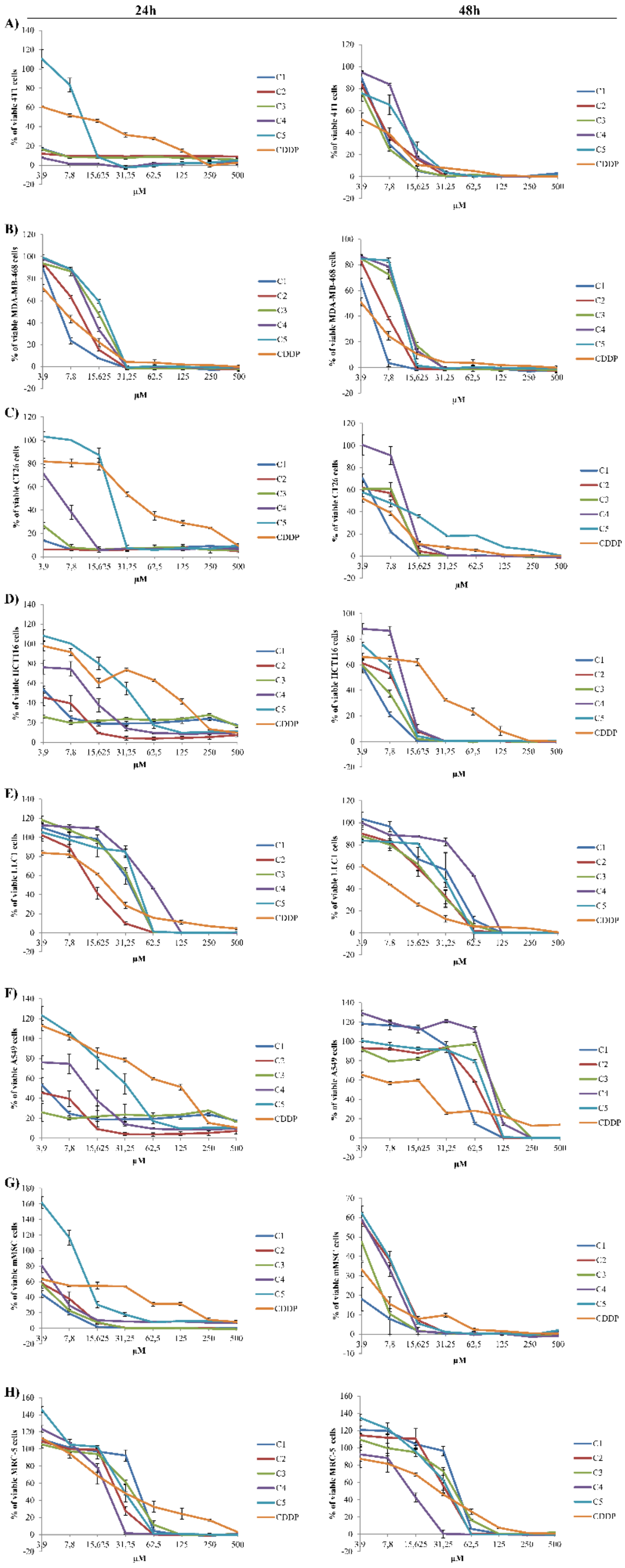
24h
48h
A)
% of viable 4T1 cells
B)
% of viable MDA-MB-468 cells
C)
% of viable CT26 cells
D)
% of viable HCT116 cells
E)
% of viable LLC1 cells
F)
% of viable A549 cells
G)
% of viable mMSC cells
H)
% of viable MRC-5 cells
3,9 7,8 15,625 31,25 62,5 125 250 500
µM
C1
C2
C3
C4
C5
CDDP

Figure S6. Dose-dependent cytotoxicity of silver(I)-complexes with S-alkyl derivatives of thiosalicylic acid. Effect of C1-C5 on viability of 4T1 (A), MDA-MB-468(B), CT26(C), HCT116(D), LLC1(E), A549(F), mMSC(G) and MRC-5(H) cells after period of incubation of 24h and 48h analyzed with the MTT assay. All data are presented as mean values ± SD from three independent experiments performed in triplicates.

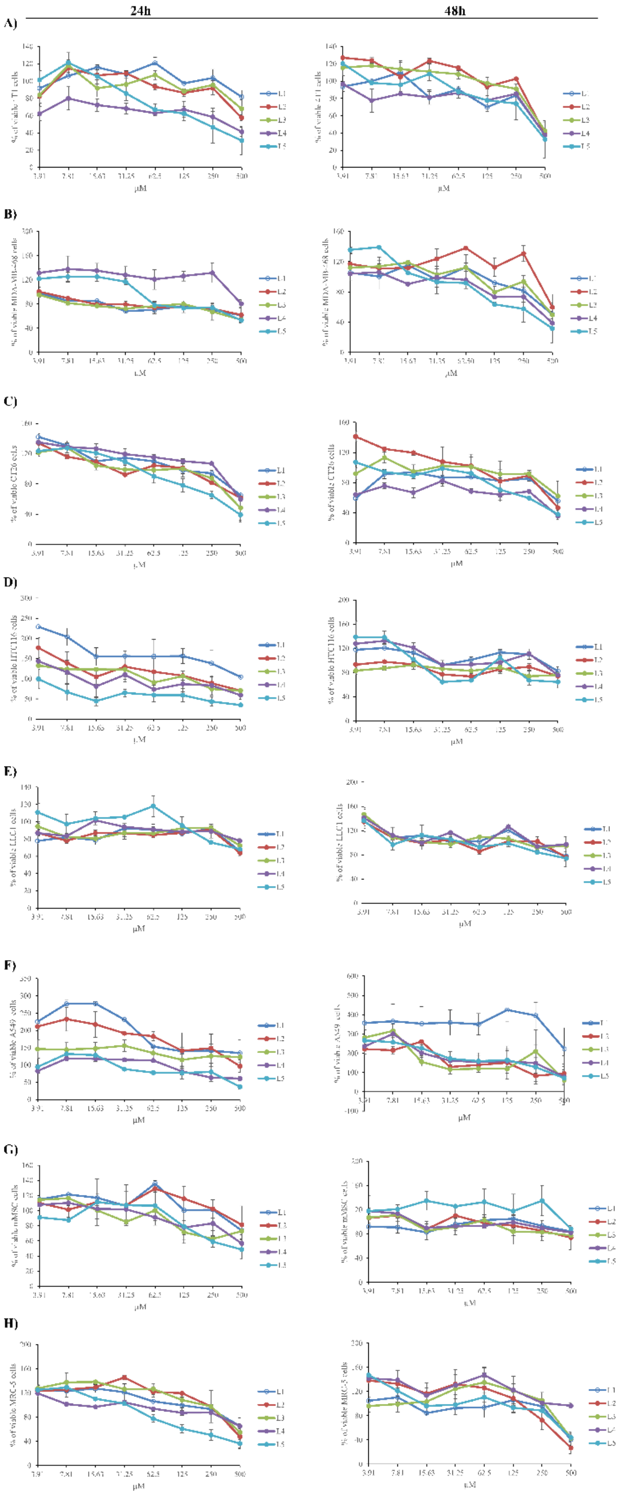
24h
48h
A)
B)
C)
D)
E)
F)
G)
H)
μM
L1
L2
L3
L4
L5

Figure S7. Dose-dependent cytotoxicity S-alkyl derivatives of thiosalicylic acid. Effect of **L1-L5** on viability of 4T1 (A), MDA-MB-468(B), CT26(C), HCT116(D), LLC1(E), A549(F), mMSC(G) and MRC-5(H) cells after period of incubation of 24h and 48h analyzed with the MTT assay. All data are presented as mean values ± SD from three independent experiments performed in triplicates.

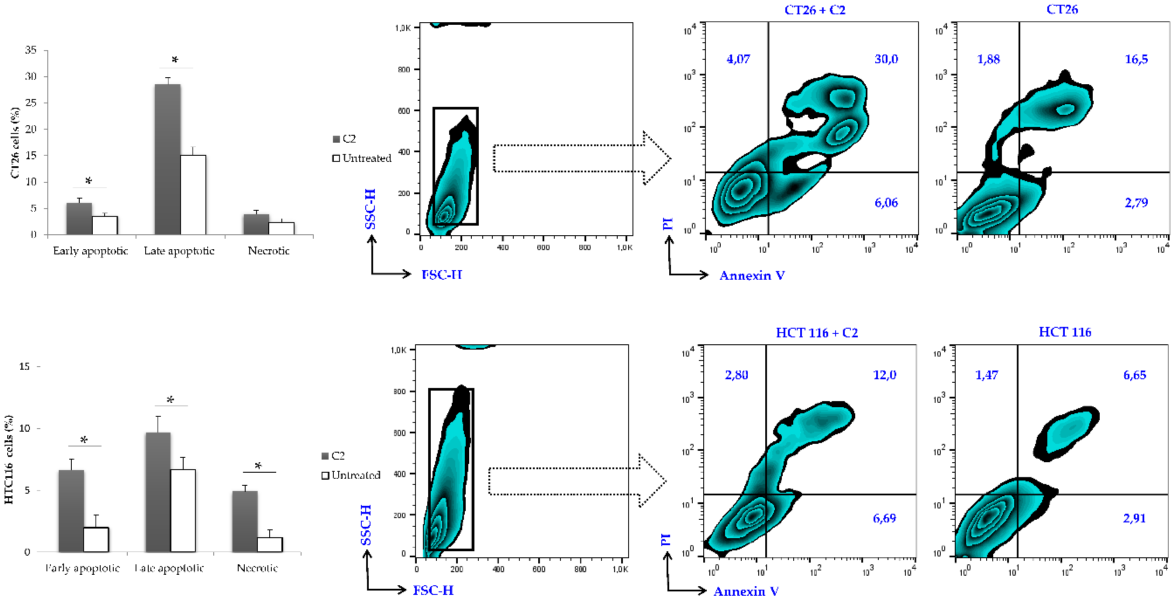

Figure S8. CT26 and HCT116 cells underwent apoptosis after **C2** treatment. Apoptotic rates of **C2** treated (0.5 μM for 24 hours) as well as untreated CT26 and HCT116 cells, were determined by flow cytometry using Annexin V (FITC) and PI double staining. The data are shown as averages ± SD of a 3 independent experiment. Mann-Whitney U test* $p<0.05$ compared with the untreated group.

IC_{50} for **C1-C5**	Kb (M^{-1}) x 10^5	
	Spearman's rho	p
4T1 24h	0.700	0.188
4T1 48h	0.100	0.873
MDA-MB-468 24h	0.900	0.037
MDA-MB-468 48h	0.700	0.188
CT26 24h	0.700	0.188
CT26 48h	1	/
HCT116 24h	0.600	0.285
HCT116 48h	-0.600	0.285
LLC1 24h	0.200	0.747
LLC1 48h	0.200	0.747
A549 24h	-0.700	0.188
A549 48h	0.700	0.188
mMSC 24h	0.700	0.188
mMSC 24h	-0.600	0.285
MRC-5 24h	-0.300	0.624
MRC-5 48h	-0.300	0.624

Figure S9. Relationship between DNA-binding constants (Kb) and IC_{50} values.
